# Chromatin profile-based identification of a novel ER-positive breast cancer subgroup with reduced ER-responsive element accessibility

**DOI:** 10.1038/s41416-023-02178-1

**Published:** 2023-02-01

**Authors:** Kohei Kumegawa, Sumito Saeki, Yoko Takahashi, Liying Yang, Tomo Osako, Tomoyoshi Nakadai, Sayuri Amino, Tetsuyo Maeda, Chikako Takahata, Seiichi Mori, Tetsuo Noda, Shinji Ohno, Takayuki Ueno, Reo Maruyama

**Affiliations:** 1 Cancer Cell Diversity Project, NEXT-Ganken Program, Japanese Foundation for Cancer Research, Tokyo, Japan; 2 Breast Surgical Oncology, Breast Oncology Center, Cancer Institute Hospital, Japanese Foundation for Cancer Research, Tokyo, Japan; 3 Project for Cancer Epigenomics, Cancer Institute, Japanese Foundation for Cancer Research, Tokyo, Japan; 4 Division of Pathology, Cancer Institute, Japanese Foundation for Cancer Research, Tokyo, Japan; 5 Project for Development of Innovative Research on Cancer Therapeutics, Cancer Precision Medicine Center, Japanese Foundation for Cancer Research, Tokyo, Japan; 6 Director’s room, Cancer Institute, Japanese Foundation for Cancer Research, Tokyo, Japan; 7 Breast Oncology Center, Cancer Institute Hospital, Japanese Foundation for Cancer Research, Tokyo, Japan

**Keywords:** Breast cancer, Breast cancer

## Abstract

**Background:**

Oestrogen receptor (ER) signalling-dependent cancer cell growth is one of the major features of ER-positive breast cancer (BC). Inhibition of ER function is a standard and effective treatment for ER-positive tumours; however, ~20% of patients with ER-positive BC experience early or late recurrence. In this study, we examined intertumour heterogeneity from an epigenetic perspective based on the hypothesis that the intrinsic difference in epigenetic states around ER signalling pathway underlies endocrine therapy resistance.

**Methods:**

We performed transposase-accessible chromatin sequencing (ATAC-seq) analysis of 42 BC samples, including 35 ER-positive(+) human epidermal growth factor receptor 2 (HER2)-negative(−) and 7 triple-negative tumours. We also reanalysed ATAC-seq data of 45 ER + /HER2 − tumours in the Cancer Genome Atlas (TCGA) BC cohort to validate our observations.

**Results:**

We conducted a comprehensive analysis of *cis*-regulatory elements (CREs) using ATAC-seq, identifying three subgroups based on chromatin accessibility profiles. We identified a subgroup of ER-positive BCs with a distinctive chromatin accessibility pattern including reduced accessibility to ER-responsive elements (EREs). The same subgroup was also observed in TCGA BC cohort. Despite the reduced accessibility to EREs, the expression of ER and potential ER target genes were not decreased in these tumours.

**Conclusion:**

Our findings highlight the existence of a subset of ER-positive BCs with unchanged ER expression but reduced EREs accessibility that cannot be distinguished by conventional immunostaining for ER. Future studies should determine whether these tumours are associated with resistance to endocrine therapy.

## Introduction

Oestrogen receptor (ER)-positive breast cancer (BC) accounts for ~70% of newly diagnosed BC cases [1]. ER signalling-dependent cancer cell growth is one of the major features of ER-positive (BC) [2], and endocrine therapies designed to block ER function, such as selective ER modulators and aromatase inhibitors [3], have been developed as adjuvant or neoadjuvant therapies for luminal BC. Although these therapies have improved prognosis for ER-positive BC, ~20% of patients who receive them experience early or late recurrence [4, 5] and require additional therapy. In current clinical practice, one of the challenges is accurately determining whether chemotherapy is necessary for a patient with ER-positive human epidermal growth factor receptor 2 (HER2)-negative BC in an adjuvant setting; the decision is based on an assessment of risk factors such as histopathology, tumour size, and the number of lymph nodes involved. However, this approach does not always accurately predict the benefit of additional treatment, and the development of more accurate biomarkers for indication to escalate or de-escalate therapy is required.

Patient stratification based on molecular profiling has been attempted for over a decade [6]. In particular, since the discovery of the intrinsic subtype in 2002 [7, 8], transcriptomic stratification has been actively pursued with some success in actual clinical practice [9]. For example, OncotypeDx assay scores the expression of 21 genes, with high scores indicating a high likelihood of future recurrence and influencing clinical decisions such as the choice of chemotherapy [10, 11]. It is possible that adding epigenetic information could improve the quality of patient stratification by providing higher precision because changes in epigenetic states may be at least partly responsible for endocrine therapy resistance [12–14]. DNA methylation has long been studied in cancer, and several studies have shown that it could be a useful indicator in the stratification of BC [8, 15–18]. However, the findings are not practically useful yet. Several studies on histone modifications, which are responsible for another layer of epigenetic regulation, have indicated the clinical significance of genetic mutations or the expressional changes of histone-modifying enzymes [19–21]. Nevertheless, no study has stratified patients with BC using genome-wide histone modification patterns, which may be because the detection of global histone modifications is not technically stable.

From this perspective, we considered the recently developed transposase-accessible chromatin sequencing (ATAC-seq) technique as the ideal solution. ATAC-seq utilises a transposase and obtains genome-wide chromatin accessibility data [22]. Given its simple experimental principle, ATAC-seq has several advantages over other epigenome analysis methods [23, 24]. For example, robust data with low variability can be obtained owing to the limited number of steps in the method, which is critical for clinical research. In one study, ATAC-seq was used to profile 410 tumour samples from 23 cancer types at The Cancer Genome Atlas (TCGA), including 75 BC samples [25]. Although these data have already been utilised in another study [26], patient stratification of BC via ATAC-seq has not been reported.

In the present study, we performed ATAC-seq analysis of BC specimens to determine whether intrinsic differences exist in epigenetic states that may not be distinguishable by conventional immunohistochemistry (IHC). We conducted a comprehensive analysis of cis-regulatory elements (CREs) using ATAC-seq, identifying three subgroups based on their chromatin accessibility profiles. Intriguingly, the ER itself was still expressed in one subgroup whereas the accessibility of EREs was reduced. The tumours in this subgroup also showed decreased accessibility of FOXA1-binding regions. The same subgroup was also observed in TCGA Breast Invasive Carcinoma (BRCA) cohort, in which oestrogen receptor 1 (*ESR1)* was transcriptionally expressed but accessibility of the EREs was reduced. Overall, we identified epigenetic diversity across ER-positive BCs without altered gene expression; therefore, the reduced accessibility of the EREs may be associated with endocrine therapy resistance and recurrence.

## Results

### Chromatin accessibility profiling of human BC samples

**Fig. 1:**
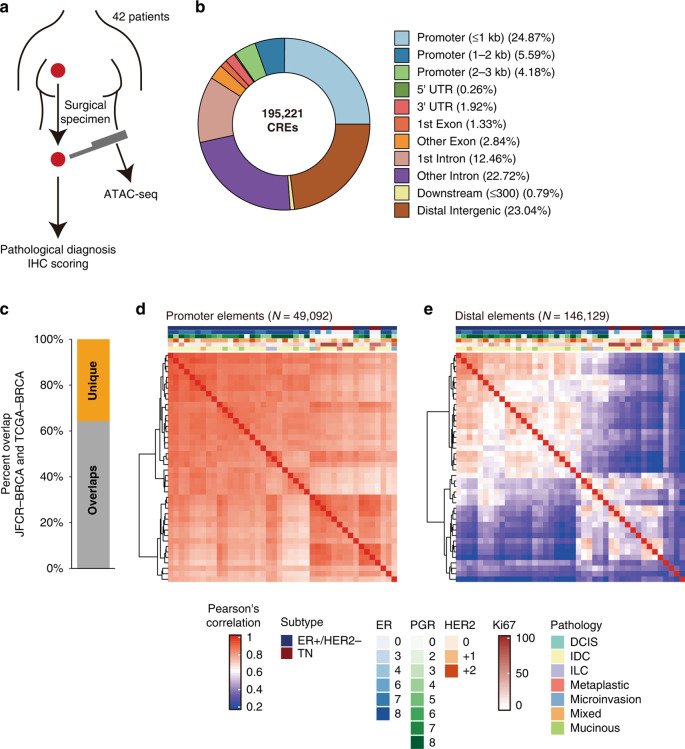
Chromatin accessibility profiling of human BC tissues. **a** Schema of sample collection and analysis. **b** Genomic features of 195,221 merged peak sets (CREs). UTR untranslated region. **c** Overlap of peaks in the JFCR–BRCA (*n* = 195,221; 42 tumours) and TCGA–BRCA (*n* = 215,920; 75 tumours) cohorts. **d**, **e** Heatmaps of Pearson correlations of ATAC-seq signal with promoters and distal elements. Patient information, including subtype, IHC scoring of ER, PgR and HER2, Ki67 scores, pathological classification, and primary or recurrent tumours, is shown above the heatmaps. Peaks around 1000 bp of the transcription start site were defined as “promoters.” Distal elements were defined as all peaks except promoters.

We profiled the epigenetic landscape using chromatin accessibility analysis of 42 prospectively collected BC samples, including 35 ER + /HER2 − and 7 triple-negative breast tumours (Table 1, JFCR–BRCA cohort). ATAC-seq requires a relatively low input [24]; therefore, we were able to use BC samples collected by core needle biopsies from surgical specimens (Fig. 1a). For all samples, the ATAC-seq data surpassed a minimum threshold of transcription start site enrichment (≥5) and exhibited a distinctive fragment size distribution with nucleosomal periodicity (Supplementary Fig. 1a–c). Using peak calling analysis, we identified and generated a merged set of 195,221 CREs (Fig. 1b**;** Supplementary Table 1). A large fraction of these CREs was found on nonexon regions, which was consistent with the findings of previous studies [25, 27]. Over 60% of the peaks overlapped between our peaks and a set of peaks identified via ATAC-seq for 75 samples in the TCGA–BRCA cohort [25] (Fig. 1c). Moreover, 32.5% overlapping CREs were distributed on promoter regions [within 1 kb of transcription start sites (TSSs)], whereas only 11.1 % of the unique CREs of the JFCR–BRCA cohort existed on promoters, highlighting the consistency and diversity of CRE activity across breast tumours (Supplementary Fig. 2a, b). CRE activity patterns were examined across tumours by calculating the Pearson’s correlation of the promoter and distal elements (Fig. 1d, e). The accessibility of promoter elements was well correlated regardless of HR status; however, distal elements showed highly specific CRE activity across tumours. This pattern, i.e. a lower correlation of the accessibility of the distal elements relative to the promoters, was also observed in the TCGA–BRCA cohort, suggesting that distal regulatory regions, such as enhancers, contributed to BC heterogeneity more substantially than promoter accessibility or gene expression.

**Table 1:** Clinicopathological characteristics of 10 patients.

Patient	Age at surgery	Menopause	Gravidity/parity	Primary or recurrent	cStage	cTNM	Histological type^a^	ER^b^	PgR^b^	HER2	Subtype classification	Ki67	Neoadjuvant
P1	76	Post	g3p2	P	I	cT1cN0M0	IDC	5 + 3	5 + 3	0	ER + /HER2−	19%	None
P3	53	Post	g0p0	P	I	cT1cN0M0	IDC	5 + 3	4 + 2	1+	ER + /HER2−	7%	None
P4	65	Post	g1p1	P	IIA	cT2N0M0	IDC	0 + 0	0 + 0	0	TNBC	85%	None
P5	79	Post	g2p0	R	I	cT1cN0M0	IDC	0 + 0	0 + 0	1+	TNBC	85%	None
P7	78	Post	g3p3	P	IIA	cT1cN1M0	IDC	0 + 0	0 + 0	0	TNBC	65%	None
P8	69	Post	g2p2	P	I	cT1micN0M0	Microinvasive	2 + 2	1 + 2	2+	ER + /HER2−	65%	None
P9	37	Pre	g0p0	P	IIA	cT2N0M0	IDC	5 + 2	5 + 2	1+	ER + /HER2−	15%	None
P11	39	Pre	g0p0	P	IIB	cT2N1M0	IDC	5 + 2	4 + 3	1+	ER + /HER2−	30%	None
P12	68	Post	g2p2	P	I	cT1cN0M0	IDC	5 + 3	5 + 3	0	ER + /HER2−	8%	None
P13	62	Post	g2p2	P	IIB	cT2N1M0	IDC	5 + 3	5 + 3	1+	ER + /HER2−	8%	None
P14	52	Pre	g2p1	P	I	cT1cN0M0	IDC	5 + 2	5 + 2	2 + ,DISH(−)	ER + /HER2−	5%	None
P15	40	Pre	g2p2	P	IIA	cT2N0M0	Mixed	5 + 2	5 + 3	1+	ER + /HER2−	7%	None
P17	55	Post	g0p0	P	IIA	cT2N0M0	Metaplastic	0 + 0	0 + 0	0	TNBC	30%	None
P18	53	Post	g0p0	P	I	cT1cN0M0	IDC	4 + 3	3 + 3	1+	ER + /HER2−	8%	None
P19	49	Pre	g1p1	P	I	cT1cN0M0	IDC	5 + 2	5 + 2	0	ER + /HER2−	15%	None
P20	54	Post	g3p2	P	IIIA	cT1cN2aM0	Metaplastic	0 + 0	0 + 0	0	TNBC	65%	None
P21	62	Post	g2p2	P	IIA	cT2N0M0	IDC	5 + 3	5 + 3	0	ER + /HER2−	10%	None
P22	51	Pre	g3p1	P	IIA	cT2N0M0	IDC	5 + 2	5 + 3	0	ER + /HER2−	45%	None
P23	69	Post	g2p1	P	I	cT1cN0M0	IDC	5 + 3	4 + 3	2+	ER + /HER2−	25%	None
P24R	54	Post	g3p2	P	I	cT1cN0M0	IDC	5 + 3	2 + 2	2 + ,DISH(−)	ER + /HER2−	3%	None
P24L	54	Post	g3p2	P	IIB	cT3N0M0	Mixed	4 + 3	0 + 0	0	ER + /HER2−	25%	None
P26	52	Pre	g1p1	P	I	cT1cN0M0	IDC	5 + 3	5 + 3	1+	ER + /HER2−	25%	None
P27	68	Post	g4p2	P	I	cT1cN0M0	IDC	5 + 3	1 + 2	0	ER + /HER2−	20%	None
P28	44	Pre	g2p2	P	IIA	cT2N0M0	IDC	3 + 1	0 + 0	0	ER + /HER2−	85%	None
P29	47	Pre	g0p0	P		TisN1M0	IDC	5 + 3	5 + 3	2 + ,DISH(−)	ER + /HER2−	25%	None
P30	70	Post	g2p2	P	IIIA	cT2N2bM0	IDC	5 + 3	4 + 2	1+	ER + /HER2−	10%	None
P31	78	Post	g2p2	P	IIIA	cT3N1M0	IDC	5 + 3	3 + 2	1+	ER + /HER2−	7%	Yes^c^
P32	68	Post	g3p3	P	IIA	cT2N0M0	IDC	5 + 3	4 + 3	0	ER + /HER2−	3%	None
P33	35	Pre	g1p1	P	I	cT1cN0M0	Mucinous	5 + 2	2 + 3	1+	ER + /HER2−	25%	None
P34	82	Post	g3p3	P	I	cT1cN0M0	IDC	5 + 2	3 + 1	0	ER + /HER2−	20%	None
P35	45	Pre	g2p1	P	I	cT1cN0M0	IDC	5 + 3	5 + 3	0	ER + /HER2−	3%	None
P36	54	Post	NA	P	IIA	cT2N0M0	IDC	5 + 3	3 + 3	1+	ER + /HER2−	40%	None
P37	91	Post	NA	P	IIA	cT2N0M0	Mucinous	5 + 3	4 + 3	1+	ER + /HER2−	8%	None
P38	63	Post	g2p2	P	I	cT1cN0M0	IDC	5 + 3	5 + 3	1+	ER + /HER2−	8%	None
P39	66	Post	g3p2	P	IIA	cT2N0M0	ILC	5 + 3	5 + 3	1+	ER + /HER2−	7%	None
P41	48	Pre	g1p1	P	I	cT1bN0M0	IDC	0 + 0	0 + 0	0	TNBC	85%	None
P44	60	Post	g2p2	P	IIA	cT2N0M0	IDC	0 + 0	0 + 0	1+	TNBC	80%	None
P49	73	Post	g0p0	P	IIA	cT2N0M0	IDC	5 + 3	5 + 3	2 + ,DISH(−)	ER + /HER2−	15%	None
P50	61	Post	g1p1	P	I	cT1cN0M0	IDC	5 + 3	0 + 0	1+	ER + /HER2−	10%	None
P54	67	Post	g0p0	P	I	cT1bN0M0	ILC	5 + 3	2 + 2	1+	ER + /HER2−	5%	None
P64	51	Post	g3p1	P	IIA	cT2N0M0	Mucinous	5 + 3	5 + 3	1+	ER + /HER2−	20%	None
P65	71	Post	g3p2	P	IIA	cT2N0M0	DCIS	1 + 2	1 + 1	2+	ER + /HER2−	15%	None

^a^*IDC* invasive ductal carcinoma, *ILC* invasive lobular carcinoma, *DCIS* ductal carcinoma in situ.

^b^IHC score based on Allred scoring system: Proportion score (ranging 0–5) plus intensity score (ranging 0–3) Proportion score (%Positive cells): 0 (0%), 1 (<1%), 2 (1–10%), 3 (11–33%), 4 (34–66%), 5 (≥67%) Intensity score: 0 (None), 1 (Weak), 2 (Intermediate), 3 (Strong).

^c^4 cycles of CEF (Cyclophosphamide, Epirubicin and 5FU) and 3wDOC (triweekly docetaxel).

### CRE-based deconvolution via single-cell ATAC-seq data analysis

**Fig. 2:**
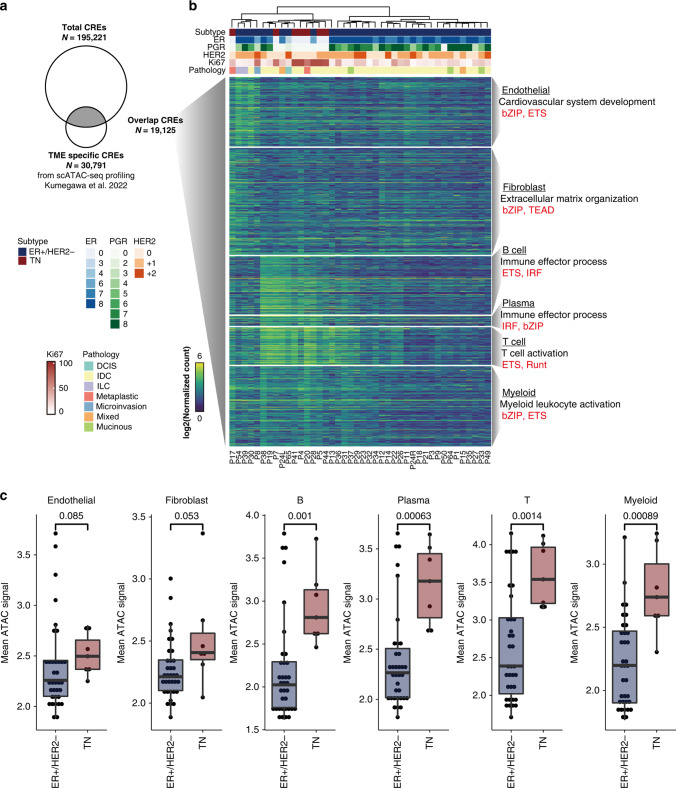
Deconvolution of ATAC-seq data using TME-specific CREs previously identified by scATAC-seq for breast tumours. **a** Venn diagram showing the overlaps between JFCR–BRCA ATAC-seq peaks (*n* = 195,221) and previously identified TME-specific CREs (*n* = 30,791). **b** Heatmap showing the chromatin accessibility of overlapping regions with each set of TME-specific CREs (endothelial, fibroblast, T cell, B cell, plasma cell and myeloid cells). The annotation above the heatmap represents patient information. Representative enrichments from GREAT GO analysis (black) and the motifs of TF families (red) are shown on the right. **c** Boxplot showing the mean ATAC-seq signal of each set of TME-specific CREs between BC subtypes. *P*-values, calculated using Wilcoxon rank sum tests, are shown.

Our ATAC-seq data also included information on cells from the tumour microenvironment (TME) because we did not physically enrich cancer cells with an antibody specific to epithelial cells. We used 30,791 TME cell-specific CREs previously identified via the single-cell (sc)ATAC-seq analysis of 16 patients with BC [28] to estimate the extent of infiltration and activity of TME cells in our ATAC-seq data. We identified 19,125 overlapping CREs between our ATAC-seq CREs and TME-specific CREs (Fig. 2a). Using GREAT gene ontology (GO) analysis [29], we identified consistent GO term enrichments of cardiovascular system development for overlaps with endothelial CREs; extracellular matrix organisation for overlaps with fibroblast CREs, and immune-associated ontology for immune CREs (Fig. 2b**;** Supplementary Table 2). Motif enrichment analysis also revealed that the motifs of the E26 transformation-specific (ETS), interferon regulatory factor and Runt transcription factor (TF) families were enriched in CREs overlapping with immune cells (Fig. 2b**;** Supplementary Table 3). Although CRE activity in epithelial- or fibroblast-specific peaks did not differ significantly between ER + /HER2 − and triple-negative breast cancer TNBC, the activity in immune-cell-specific peaks was higher in the TNBC samples than in the ER + /HER2 − samples (Fig. 2b, c). These results were consistent with a previous study revealing the enrichment of tumour-infiltrating leucocytes in ER-negative BCs [30]. Thus, our method utilising scATAC-seq data could predict the TME activity of each tumour.

### Chromatin accessibility differences between ER + /HER2 − and TNBC

**Fig. 3:**
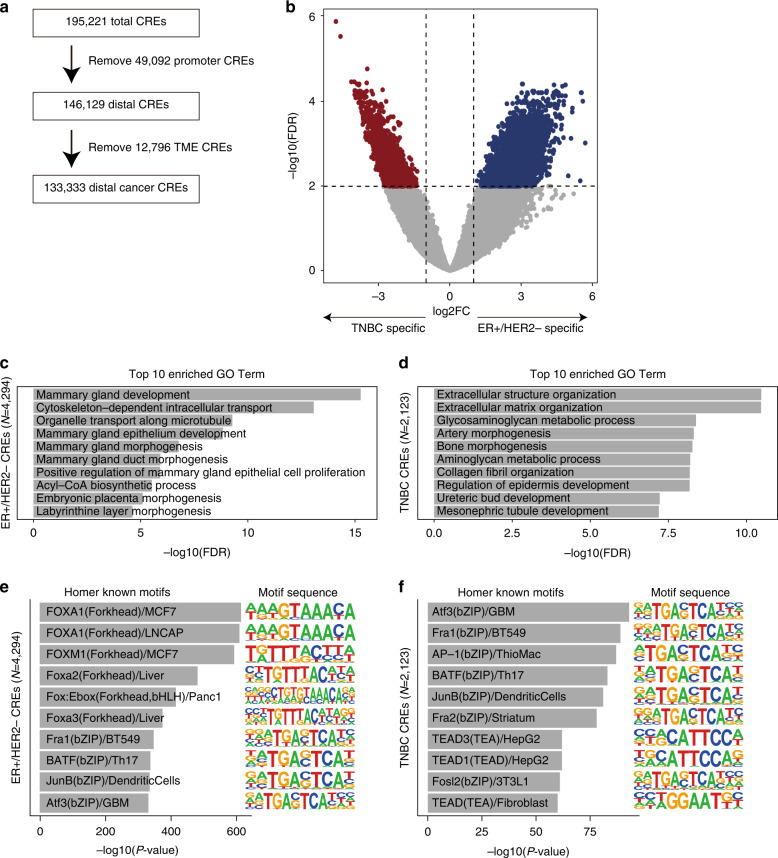
Difference in chromatin accessibility between ER + /HER2− tumours and TNBC. **a** Flow chart of peak filtering. The number of cancer CREs (*n* = 133,333) was identified by subtracting the number of TME-specific peaks (*n* = 12,796) from the total number of distal elements (*n* = 146,129). **b** Volcano plot showing the differential accessibility analysis of CREs between ER + /HER2− and TNBC. Significantly different CREs are coloured red (TNBC-specific) or blue (ER + /HER2 − -specific). **c**, **d** Bar plots of GO enrichment obtained via GREAT analysis of ER + /HER2 − -specific peaks (**c**) and TNBC-specific peaks (**d**). **e**, **f** Bar plots of the motif enrichment significance (*P*-value) of Homer known motifs for ER + /HER2 − -specific peaks (**e**) and TNBC-specific peaks (**f**). Known motif sequences are shown on the right.

To assess intertumour heterogeneity in chromatin accessibility, we defined “distal Cancer CREs” by performing the following steps: (1) 49,092 promoter elements (1 kb upstream from TSSs) were filtered out because the chromatin accessibility in the distal element exhibited diversity across tumours, and (2) 19,787 TME-specific CREs were filtered out. The remaining 133,333 CREs were defined as “distal cancer CREs” (Fig. 3a). Using the “distal Cancer CREs”, we first examined the differences in the chromatin accessibility signatures of ER + /HER2 − and TNBC, and identified 4294 ER + /HER2 − CREs and 2123 TNBC CREs (log2FC > 1 and FDR < 0.01) (Fig. 3b). The ER + /HER2 − CREs were associated with mammary gland development and mammary epithelial cell proliferation (Fig. 3c), whereas the TNBC CREs were associated with extracellular structure organisation and the morphogenesis of stromal tissues (Fig. 3d).

Motif enrichment analysis revealed that the binding motifs of FOXA1, which is a luminal-lineage TF were most enriched in ER + /HER2 − CREs (Fig. 3e**;** Supplementary Table 4). The ERE motif was also highly enriched in ER + /HER2 − CREs (rank = 33, *P* = 10^−26^) (Supplementary Table 4). In TNBC CREs, the motifs of activator protein 1 (AP-1) and TEA domain family TFs were enriched (Fig. 3f; Supplementary Table 5). Given that motif analysis using all ATAC-seq reads, i.e. nucleosome-free and nucleosome-containing reads, might generate artifacts [31], we also performed motif analysis using only nucleosome-free reads. The differential analysis using only nucleosome-free reads revealed 2415 ER + /HER2 − CREs and 607 TNBC CREs, and the results of the motif analysis were generally consistent (Supplementary Fig. 3a–c). These results suggest that ER + /HER2 − and TNBC have distinct chromatin accessibility signatures.

### ER + /HER2 − tumours exhibit three distinct chromatin accessibility clusters

We next focused on the diversity of chromatin accessibility across only ER + /HER2 − tumours. First, we identified 3516 (2.64%) commonly accessible CREs in 35 ER + /HER2 − tumours (median accessibility ≥3 and variance ≤0.5; Supplementary Fig. 4a). These CREs were associated with mRNA metabolic processes, and the CTCF binding motif was the most highly enriched (Supplementary Fig. 4b, c). We did not find high enrichment of FOXA1 (rank = 64, *P* = 10^-10^) or ERE (not significant) motifs in these CREs (Supplementary Table 6), indicating that ER + /HER2 − tumours exhibit divergent chromatin accessibility patterns of the ERE and FOXA1-binding regions.

**Fig. 4:**
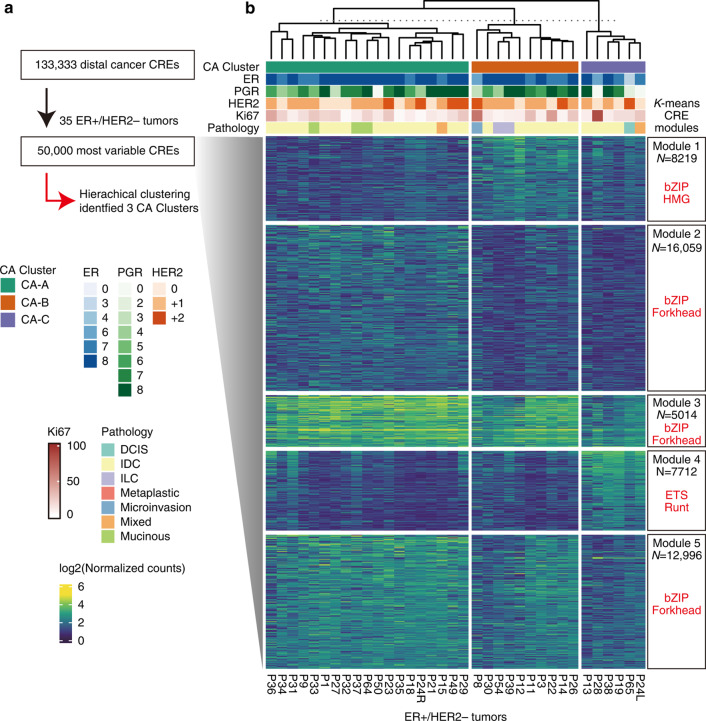
Chromatin accessibility-based classification of ER + /HER2 − tumours and the *cis*-regulatory landscape. **a** Flow chart of tumour classification. Across 35 ER + /HER2 − tumours, the top 50,000 variable CREs were selected from 133,333 distal cancer CREs, after which hierarchical clustering was performed. **b** Heatmap showing the chromatin accessibility of k-means clusters of the top 50,000 variable CREs (Modules 1–5). The annotation above the heatmap represents chromatin accessibility clusters and patient information. Boxes on the right represent the number of CREs and TF motif enrichment in each peak set.

Next, we classified 35 ER + /HER2 − tumours using hierarchical clustering based on the 50,000 most variable distal cancer CREs, identifying three distinct chromatin accessibility clusters: CA-A, CA-B and CA-C (Fig. 4a). CA-C tumours have relatively low IHC scores for ER and progesterone receptor (PgR), although other clinicopathological features such as age, menopausal status, Ki67 and histological type were not associated with the chromatin accessibility clusters (Fig. 4a**;** Supplementary Fig. 5). Principal component analysis revealed that CA-C tumours had a similar chromatin accessibility profile to that of TNBC (Supplementary Fig. 6). The accessibilities of immune-cell-specific CREs in CA-C were significantly higher than those in CA-A and CA-B, suggesting that CA-C had a similar pattern of immune-cell enrichment to that of TNBC (Fig. 2**;** Supplementary Fig. 7). Interestingly, CA-B had higher accessibility of endothelial- and fibroblast-specific CREs compared with those of the other clusters (Supplementary Fig. 7).

To characterise the chromatin accessibility clusters via the cis-regulatory landscape, we conducted k-means clustering of the top 50,000 variable peaks, classifying them into five distinct sets of CREs (Modules 1–5; Fig. 4b). Module 1 with the enrichment of Sox [high mobility group (HMG)] motifs was accessible in CA-B, Module 2 with FOXA1 motif enrichment was accessible in CA-A, Module 3 with the FOXA1 motif was highly active in CA-A and CA-B, Module 4 was accessible in CA-C, and Module 5 with the AP-1 and FOX family motifs was enriched in CA-A and CA-B (Fig. 4b**;** Supplementary Table 7). The ETS and Runt family motifs were enriched in Module 4, demonstrating the TNBC-like features [32] of CA-C tumours.

Taken together, these results suggest that CA-A and CA-B have a luminal BC signature of regulatory elements, whereas CA-C has a TNBC regulatory element pattern. Moreover, CA-B exhibited a distinctive epigenetic state with Sox family TF motif enrichment.

### CA-C exhibited the distinct chromatin signatures of ETS motif enrichment

**Fig. 5:**
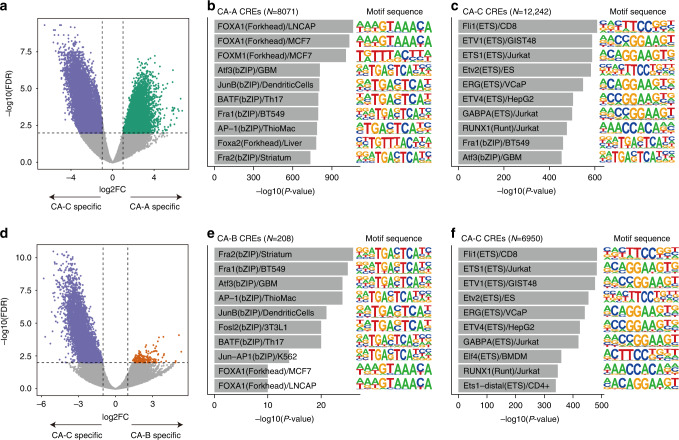
Difference in chromatin accessibility between CA-C and other tumours. **a** Volcano plot showing the differential accessibility analysis of CREs between CA-C and CA-A tumours. Significantly different CREs are coloured according to the clusters corresponding to Fig. 4b. **b**, **c** Bar plots of the motif enrichment significance (*P*-value) of Homer known motifs for CA-A–specific peaks (**b**) and CA-C–specific peaks (**c**). Known motif sequences are shown on the right. **d** Volcano plot showing the differential accessibility analysis of CREs between CA-C and CA-B tumours. Significantly different CREs are coloured according to the clusters corresponding to Fig. 4b. **e**, **f** Bar plots of the motif enrichment significance (*P*-value) of Homer known motifs for CA-B–specific peaks (**e**) and CA-C–specific peaks (**f**). Known motif sequences are shown on the right.

Although CA-C tumours were ER + /HER2 − , they possessed a chromatin accessibility pattern like that of TNBC samples. To characterise CA-C tumours, we conducted differential accessibility analysis of CA-C and CA-A as well as CA-C and CA-B. Comparison of CA-C and CA-A, we identified 12,242 CA-C-specific and 8071 CA-A-specific CREs (log2FC > 1 and FDR < 0.01; Fig. 5a). Motif enrichment analysis of these specific CREs revealed significant ETS family motif enrichment in CA-C-specific CREs and FOXA1 motif enrichment in CA-A-specific CREs (Fig. 5b, c**;** Supplementary Tables 8 and 9). We also identified 6950 CA-C-specific and 208 CA-B-specific CREs compared with CA-C and CA-B (log2FC > 1 and FDR < 0.01; Fig. 5d). As well as the comparison between CA-C and CA-B, CA-C-specific CREs contained significant enrichment of ETS family motifs (Fig. 5e, f**;** Supplementary Tables 10 and 11). The series of motif enrichment analysis were consistent with similar analyses using nucleosome-free reads (Supplementary Fig. 8). Collectively, these results suggest that CA-C tumours exhibit distinct chromatin accessibility associated with the ETS TF family involved in BC progression [33, 34].

### CA-B exhibited high-ER IHC score but low ERE accessibility

**Fig. 6:**
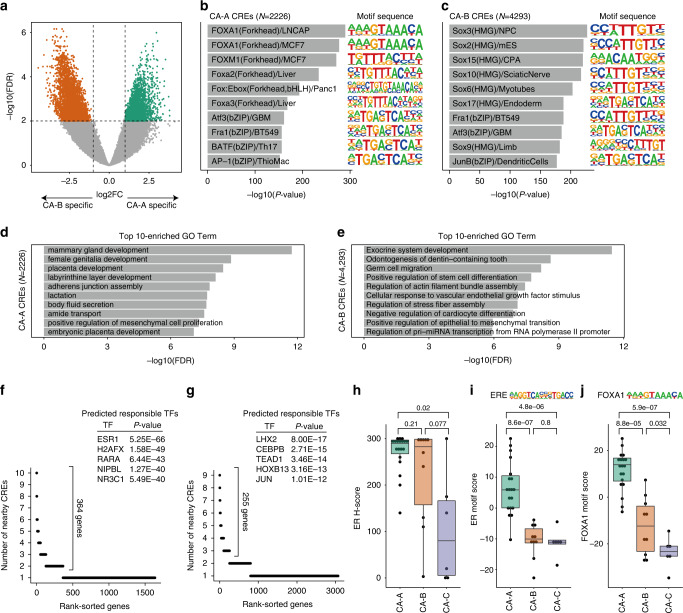
Difference in chromatin accessibility between CA-A and CA-B. **a** Volcano plot showing the differential accessibility analysis of CREs between CA-A and CA-B tumours. Significantly different CREs are coloured according to the clusters corresponding to Fig. 4b. **b**, **c** Bar plots of the motif enrichment significance (*P*-value) of Homer known motifs for CA-A–specific peaks (**b**) and CA-B–specific peaks (**c**). Known motif sequences are shown on the right. **d**, **e** Bar plots of GO enrichment obtained using GREAT analysis of CA-A–specific peaks (**d**) and CA-B–specific peaks (**e**). **f**, **g** Dot plots representing the number of nearby CA-A–specific peaks (**f**) and CA-B–specific peaks (**g**) per gene. Each dot represents a different gene. Inset tables showing the top five predicted responsible TFs regulating the indicated genes identified via LISA analysis. **h** Boxplot representing the ER IHC score (H-score) of each tumour. *P*-values, calculated via Student’s *t*-test, are shown. **i**, **j** Boxplots representing the ER motif score (**i**) and FOXA1 motif score (**j**) of each chromatin accessibility cluster. Motif sequences are shown above the boxplots. *P*-values, calculated via Student’s *t*-test, are shown.

The chromatin accessibility clusters CA-A and CA-B exhibited similar *cis*-regulatory landscapes; however, for some CRE modules, the accessible patterns differed between CA-A and CA-B (Fig. 4b). To identify the different regulatory signatures between CA-A and CA-B, we performed differential analysis, identifying 2226 CA-A-specific and 4293 CA-B-specific CREs (log2FC > 1 and FDR < 0.01; Fig. 6a). Motif enrichment analysis revealed that the FOXA1 motif was the most highly enriched in CA-A-specific CREs, whereas Sox motifs were the most highly enriched in CA-B-specific CREs (Fig. 6b, c**;** Supplementary Tables 12 and 13). FOXA1 motif enrichment in CA-A-specific CREs and Sox motif enrichment in CA-B-specific CREs were validated by performing an analysis using nucleosome-free reads (Supplementary Fig. 9a–c). A previous study in which TCGA–BRCA ATAC-seq data were reanalysed revealed that the FOXA1 motif was more enriched in ER + /HER2 − invasive ductal carcinoma (IDC) tumours than in ER + /HER2 − invasive lobular carcinoma (ILC) tumours [26]. The JFCR–BRCA cohort included two ILC tumours, which were all assigned to CA-B; therefore, we conducted differential analysis between CA-A and CA-B but only for IDC tumours. In our cohort, CA-A IDC tumours exhibited significantly higher FOXA1 motif enrichment than CA-B IDC tumours (Supplementary Fig. 10a, b), suggesting that the motif enrichment results were independent of sample histology. Using GREAT analysis, we identified characteristic GO term enrichments of mammary gland development and female genitalia development in CA-A-specific CREs and exocrine system development, germ cell migration, and positive regulation of stem cell differentiation in CA-B-specific CREs (Fig. 6d, e), suggesting CA-A-specific CREs were associated with the development of the luminal epithelium and reproductive system, whereas CA-B-specific CREs were associated with mesenchymal or stemness features. Epigenetic Landscape In Silico deletion Analysis (LISA) were used to infer transcriptional regulators [35] According to LISA analysis, the genes nearby CA-A-specific CREs (364 genes; number of nearby CREs ≥2; Supplementary Table 14) were predicted to be regulated by ER (Fig. 6f), suggesting that CA-A-specific CREs could be regulatory regions of ER target genes. The genes nearby CA-B-specific CREs (255 genes; number of nearby CREs ≥3; Supplementary Table 15) were predicted to be regulated by LIM homeobox 2 (LHX2), CCAAT enhancer-binding protein (CEBP)B and TEAD1 (Fig. 6g), which was consistent with TEAD1 motif enrichment in the CA-B-specific nucleosome-free peaks (Supplementary Fig. 9c).

ER expression was evaluated in detail by pathologically reanalyzing tumour samples using H-scoring [36]. Consistent with IHC scoring using the Allred score, ER H-scoring did not differ significantly between CA-A and CA-B (Fig. 6h). The ER motif enrichment score was also calculated for each tumour using ChromVAR [37]. Unlike ER expression, the ER motif score was significantly lower in CA-B than in CA-A (Fig. 6i**;** Supplementary Fig. 11a). Intriguingly, the expression of the PgR, which is a classic downstream target of ER, was not lower in CA-B despite lower ER motif accessibility (Supplementary Fig. 11b). Consistent with the motif analysis of each specific CRE, CA-B exhibited a lower FOXA1 motif score and higher Sox3 motif score than those of CA-A (Fig. 6j**;** Supplementary Fig. 11a).

Taken together, these findings indicate that CA-A and CA-B have similar ER expression levels (as indicated by the ER IHC score); however, the accessibility of EREs was significantly lower in tumours with CA-B, suggesting that ER + /HER2 − tumours with CA-B are epigenetically different from ER + /HER2 − tumours with CA-A in terms of ER-associated regulatory landscape.

### Reduced accessibility of EREs without a change in ER expression in a subset of luminal BCs in the TCGA–BRCA cohort

**Fig. 7:**
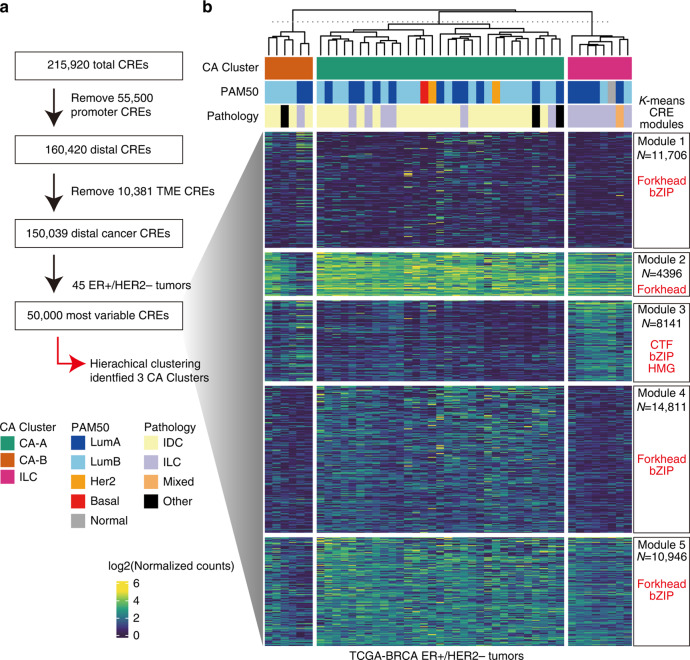
Chromatin accessibility-based classification of TCGA–BRCA ER + /HER2 − tumours. **a** Flow chart of peak filtering and tumour classification. The distal cancer CREs (*n* = 150,039) were identified by removing TME-specific peaks (*n* = 10,381) from the distal elements (*n* = 160,420). Across 45 ER + /HER2 − tumours, the top 50,000 variable CREs were selected from 150,039 distal cancer CREs, after which hierarchical clustering was performed. **b** Heatmap showing the chromatin accessibility of the k-means clusters of the top 50,000 variable CREs (Modules 1–5). The annotation above the heatmap represents chromatin accessibility clusters, PAM50 classification and histological type. Boxes on the right represent the number of CREs and TF motif enrichment in each peak set.

To validate our observations of distinct BC chromatin accessibility clusters, we reanalysed the ATAC-seq data of the TCGA–BRCA cohort [25]. First, we identified 150,039 distal cancer CREs by filtering out promoter elements (*n* = 55,500) and TME-specific CREs identified via our scATAC-seq analysis (*n* = 10,381) (Fig. 7a). Next, we classified 45 ER + /HER2 − tumours via hierarchical clustering based on the 50,000 most variable distal cancer CREs, identifying 3 distinct chromatin accessibility clusters: CA-A, CA-B and ILC-enriched (Fig. 7b). CA-A contains 11 Luminal A, 17 Luminal B, 2 HER2 tumours as well as 1 basal tumours; CA-B contains 2 Luminal A and 4 Luminal B tumours; and ILC-enriched contains 5 Luminal A, 2 Luminal B tumours as well as 1 normal tumour (Fig. 7b). These result suggest that the chromatin clusters were not associated with Prediction Analysis of Microarray 50 (PAM50) intrinsic subtype [9] based on the transcriptional output. Principal component analysis also indicated that each chromatin cluster possessed a unique chromatin accessibility profile and was not associatiated with PAM50 classification (Supplementary Fig. 12). As well as the analysis of JFCR–BRCA ER + /HER2 − tumours, we conducted k-means clustering of the top 50,000 variable peaks by classifying them into five distinct sets of CREs (Modules 1–5; Fig. 7b). Module 3, exhibiting the enrichment of NF1 (CTF), AP-1 (bZIP) and Sox (HMG) motifs, was relatively accessible in CA-B and ILC-enriched; other modules with FOXA1 motif enrichment were accessible in CA-A (Fig. 7b**;** Supplementary Table 16).

**Fig. 8:**
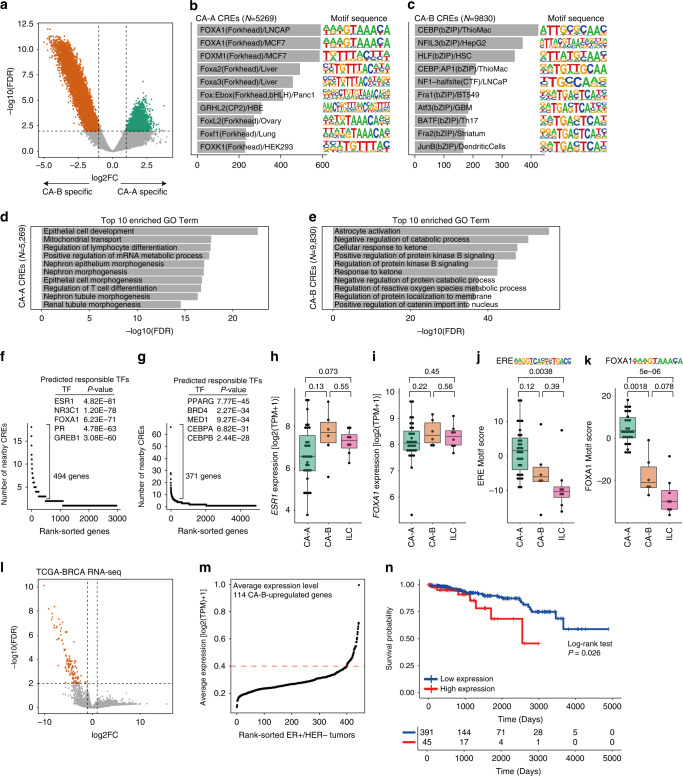
Difference in chromatin accessibility between TCGA CA-A and CA-B tumours. **a** Volcano plot showing the differential accessibility analysis of CREs between TCGA CA-A and CA-B tumours. Significantly different CREs are coloured according to the clusters corresponding to Fig. 7b. **b**, **c** Bar plots of the motif enrichment significance (*P*-value) of Homer known motifs for CA-A–specific peaks (**b**) and CA-B–specific peaks (**c**). Known motif sequences are shown on the right. **d**, **e** Bar plots of GO enrichment obtained using GREAT analysis of CA-A–specific peaks (**d**) and CA-B–specific peaks (**e**). **f**, **g** Dot plots representing the number of nearby CA-A–specific peaks (**f**) and CA-B–specific peaks (**g**) per gene. Each dot represents a different gene. Inset tables show the top five predictions of the TFs responsible for regulating the indicated genes according to LISA analysis. **h** Boxplot representing RNA-seq *ESR1* expression. *P*-values, calculated via Wilcoxon rank sum tests, are shown. **i** Boxplot representing ER motif scores. *P*-values, calculated via Student’s *t*-test, are shown. **j** Boxplot representing RNA-seq *FOXA1* expression. *P*-values, calculated via Wilcoxon rank sum tests, are shown. **k** Boxplot representing FOXA1 motif scores. *P*-values, calculated via Student’s *t*-test, are shown. **l** Volcano plot showing differential expression analysis of the RNA-seq data of TCGA CA-A and CA-B. **m** Dot plot showing the average expression levels of TCGA–BRCA ER + /HER2 − tumours (RNA-seq data; 436 tumours). Red dashed line represents the cutoff value (0.4) in survival analysis. **n** Kaplan–Meier plot of patients with high (*n* = 45) and low expression (*n* = 391) levels of upregulated genes in CA-B.

We conducted differential analysis to determine whether CA-A/B in TCGA–BRCA cohort had chromatin accessibility features in common with CA-A/B in the JFCR–BRCA cohort, identifying 5269 CA-A-specific and 9830 CA-B-specific CREs (log2FC > 1, FDR < 0.01; Fig. 8a). Motif analysis revealed that the FOXA1 and CEBP motifs were the most highly enriched in CA-A-specific and CA-B-specific CREs, respectively (Fig. 8b, c**;** Supplementary Tables 17 and 18). Using GREAT analysis, we identified the GO term enrichments of epithelial cell development in CA-A-specific CREs and astrocyte activation, negative regulation of catabolic process, and cellular response to ketone in CA-B-specific CREs (Fig. 8d, e). LISA analysis revealed that genes nearby CA-A-specific CREs (494 genes; number of nearby CREs ≥3; Supplementary Table 19) were predicted to be regulated by ER (Fig. 8f); whereas the genes nearby CA-B-specific CREs (371 genes; number of nearby CREs ≥5; Supplementary Table 20) were predicted to be regulated by peroxisome proliferator–activated receptor gamma (PPARG), bromodomain-containing protein 4 (BRD4), mediator complex subunit 1 (MED1), CEBPA and CEBPB (Fig. 8g). We also found that CA-A-specific or CA-B-specific CREs present between JFCR–BRCA and TCGA–BRCA were equally and significantly overlapped respectively (CA-A CREs: 404 overlaps, *P*-value = 1e-678, CA-B CREs: 360 overlaps, *P*-value = 1e-376: Supplementary Fig. 13a, b). Both sets of CA-A-specific CREs in TCGA and JFCR significantly overlapped publicly available FOXA1 ChIP-seq peaks in the ER + /HER2 − cell lines T-47D and MCF-7, whereas both sets of CA-B-specific CREs in TCGA and JFCR were less overlapped the FOXA1 peaks (Supplementary Fig. 13c–h). In JFCR–BRCA tumours, we confirmed that both CA-A and CA-B exhibited high expression levels of ER using IHC (Fig. 6h). To validate the ER expression state in TCGA–BRCA tumours, we used RNA-seq data for the corresponding samples of the ATAC-seq data. Consistent with the JFCR–BRCA, *ESR1* expression levels were almost the same in TCGA CA-A and CA-B (Fig. 8h). We also evaluated *FOXA1* expression, confirming that no difference existed between chromatin accessibility clusters (Fig. 8i). The ChromVAR motif scores of ER and FOXA1 were relatively lower in CA-B than in CA-A (ER: *P* = 0.12, FOXA1: *P* = 0.0018; Fig. 8j, k). These results suggest that the CA-B tumours in both cohorts can be defined as follows: (1) *ESR1* and *FOXA1* are expressed at both RNA and protein levels; (2) fewer accessible EREs and FOXA1-regulated CREs are associated with luminal features. These findings suggest that the ER-responsive cistrome in a subset of ER + /HER2 − BCs is reprogramed without changing the transcriptional output.

Finally, we conducted survival analysis based on the chromatin accessibility clusters in the TCGA–BRCA cohort. Owing to the small size of the patient cohort, we did not find significant associations between chromatin clusters and patient outcome (Supplementary Fig. 14). To overcome this limitation, we performed differential analysis of CA-A and CA-B based on the RNA-seq data of corresponding tumours, identifying 114 upregulated genes for CA-B (Fig. 8l**;** Supplementary Table 21) as the possible surrogate markers of CA-B (Fig. 8l**;** Supplementary Table 21). No GO terms were significantly enriched (adjusted *P*-value < 0.05) for these genes as well as few genes of them overlapped the ENCODE ER target genes (1 gene) or FOXA1 target genes (7 genes). Also, few genes were overlapped with the genes proximal to CA-A (7 genes) or CA-B-specific peaks (9 genes) (Supplementary Table 21). Thus, these 114 genes did not seem to represent a functional feature of CA-B or to be directly related to CA-B-specific cistrome, but nevertheless it still had potential to surrogate CA-B subgroup specificity and was used in the following survival analysis. Although the average expression levels of these genes in the most of ER + /HER2 − tumours were low, high expression levels were found in a subset of tumours (45 of 436 ER + /HER2 − tumours; cutoff = 0.4; Fig. 8m). The patients that exhibited high expression of the CA-B markers exhibited lower overall survival (Fig. 8n), implying that the CA-B chromatin accessibility profile was associated with a poor outcome in ER + /HER2 − tumours.

## Discussion

In this study, we performed ATAC-seq analysis of BC specimens and observed intertumour epigenetic heterogeneity, which cannot be distinguished by gene expression, in ER + /HER2 − BCs. We identified a subset of ER-positive tumours with reduced ERE accessibility but sustained ER expression at both the RNA and protein level. Previous studies using single-cell or bulk assays to examine RNA expression, chromatin accessibility, histone modification, and DNA methylation [38–42] have revealed transcriptional and epigenetic diversity among patients with BC; however, a dissociated state between transcription and the epigenome has not been reported. We categorised distal cancer CREs using a set of TME-derived CREs previously reported in our scATAC profile of primary breast tumours [28] identifying a subset of ER-positive tumours with sustained ER expression (at both the RNA and protein levels). We observed the reduced accessibility of EREs in two independent cohorts (42 and 75 samples in the JFCR–BRCA and TCGA–BRCA cohorts, respectively), suggesting that the current classification system for ER-positive tumours based on gene expression, particularly the expression of ER, PgR and Ki67, is not sufficient for understanding the nature of BC.

In terms of the stratification of ER-positive BC patients, gene expression profile-based scoring, such as Oncotype DX, has been used in actual clinical practice [6]. In addition, several attempts to stratify patients using DNA methylation have also been reported. For example, Fang et al. focused on the B-CIMP phenotype, indicating that even among patients with ER-positive BCs, those that were CIMP-negative exhibited more metastatic disease and worse prognosis [16]. Unfortunately, in the present study, it was not possible to determine whether the CA-A, CA-B, and CA-C classifications are associated with the Oncotype DX scores and CIMP phenotype described above. If the CA-A and CA-B classifications are correlated with the Oncotype DX scores, the present results may provide insights into the biology underlying Oncotype DX. However, if these classifications are not associated with the Oncotype DX scores, information on chromatin accessibility could provide an entirely new perspective on stratification. We will investigate these possibilities in a follow-up study.

The EREs with reduced accessibility were also enriched in FOXA1-binding motifs (Fig. 6). FOXA1 is a luminal-lineage TF [43]; therefore, decreasing the accessibility of these elements may result in the dedifferentiation of luminal cancer cells into a basal or mesenchymal state, leading to endocrine resistance and metastasis. We observed BCs with reduced ERE accessibility (CA-B) in both the JFCR–BRCA and TCGA–BRCA cohorts (Figs. 2 and 5). Interestingly, the enriched motifs in CA-B-specific peaks differed between the two cohorts, whereas the enriched motifs in CA-A-specific peaks were common, including FOXA1, AP-1 and ERE. In the JFCR–BRCA cohort, Sox TF binding motifs were enriched in CA-B peaks (Fig. 4c). The Sox family is associated with a pluripotent cell state [44] and often promotes cancer dedifferentiation and metastasis [45], suggesting that tumours with CA-B lose luminal features and acquire potentially metastatic characteristics. In the TCGA–BRCA cohort, NFI TF motifs were enriched in CA-B-specific peaks (Fig. 5f). NFIB was previously reported as a TF that binds to the ER and promotes an oestrogen-independent phenotype by activating fibroblast growth factor receptor 2 signalling [46], which activates the expression of endoplasmic reticulum oxidoreductase 1 alpha and enhances hypoxia-inducible factor 1 alpha–vascular endothelial growth factor A-mediated angiogenesis and metastasis [47]. Both enriched TF motifs were associated with tumour progression or metastasis, suggesting that the decreased accessibility of EREs is a common phenomenon in the two cohorts, although there may be different underlying mechanisms.

In conclusion, we identified a subgroup of ER + /HER2 − with reduced ERE accessibility. The subgroup may represent diversity of the ER gene regulatory programme without the modification of gene expression, and may be associated with endocrine therapy resistance. However, our data are based on fresh samples collected prospectively to obtain high-quality ATAC-seq data and are not accompanied by clinical information. Therefore, we cannot directly examine the association between chromatin accessibility patterns and endocrine resistance or prognosis. However, in the future, when the protocol for ATAC-seq experiments using fresh frozen samples improves and more stable data are obtained, we will be able to analyse archived samples with accompanying clinical information, and then we will be able to clarify the clinical significance of this subgroup with reduced ERE accessibility.

## Methods

### Clinical specimens

BC specimens were obtained by core needle biopsy of surgically removed tumours. Specimens were dissociated into single cells using a MACS Tumor Dissociation Kit and a gentleMACS Dissociator (Miltenyi Biotec) according to the manufacturer’s instructions. Cells were cryopreserved in Bambanker freezing medium (Nippon Genetics) for ATAC-seq analysis.

### ATAC-seq library preparation

Cryopreserved cells were thawed and used for ATAC-seq analysis. ATAC-seq libraries were prepared according to the Omni-ATAC protocol [24]. Briefly, 50,000 cells were lysed to release the nuclei and subjected to a transposition reaction. The transposed fragments were pre-amplified, quantitated by real-time PCR, and then amplified again. Prepared libraries were sequenced on the Illumina NextSeq 550 platform (Illumina) with paired-end reads (read 1, 75 bp; index 1, 8 bp; index 2, 8 bp, read 2, 75 bp).

### ATAC-seq data analysis—processing and alignment

For ATAC-seq data processing and alignment, PEPATAC pipeline (http://code.databio.org/PEPATAC/) was used. Fastq files were trimmed to remove Illumina Nextera adapter sequence using Skewer [48] with “-f sanger -t 20 -m pe -x” options. After trimming, sequencing quality validation was performed using FastQC [49]. For removing reads from chrM or repeat sequences, pre-alignments to eliminate reads that would map to these regions using Bowtie2 [50] with “-k 1 -D 20 -R 3 -N 1 -L 20 -I S,1,0.50 -X 2000 --no-mixed --no-discordant” options. Filtered reads were aligned to the hg38 human reference genome using Bowtie2 with “--very-sensitive -X 2000 -no-mixed --no-discordant” options. For removing duplicates, Picard (http://broadinstitute.github.io/picard/) MarkDuplicates tool was used with “VALIDATION_STRINGENCY = LENIENT REMOVE_DUPLICATES = true” options. Final aligned, de-duplicated bam files were used in all downstream analysis.

### ATAC-seq data analysis—quality check

For quality estimation of each ATAC-seq profiles, enrichment of ATAC-seq accessibility at transcription start sites (TSSs) and fragment length distribution was used. Bam files were import as Genomic Ranges object in R using “scanbam” command of Rsamtools and corrected by an offset to the read start (“+” stranded +4 bp, “−” stranded −5 bp). For TSS enrichment profiling, each TSS (TSS position were obtained by transcripts(TxDb) command from “TxDb.Hsapiens.UCSC.hg38.knownGene” package) was extended 2000 bp in each direction and then overlapped with the insertions, i.e. either end of a fragment, using “findOverlaps()”. We then calculated the distance between the insertions and the strand-corrected TSS. After that, the number of insertions in each single-base bin was summed. For normalisation of the values, the accessibility at each position ±1900–2000 bp from the TSS. The final TSS enrichment was the maximum enrichment value within ±50 bp of the TSS after smoothing with a rolling mean every 51 bp. For making fragment length distribution, the width of each fragment was plotted.

### ATAC-seq data analysis—peak calling and making a counts matrix

For generation of high-quality peak set from 42 ATAC-seq profiles, we conducted analysis following steps described in ref. [25] (1) peak calling on the Tn5-corrected single-base insertions from each tumour was conducted using MACS2 [51] with “--shift -75 --extsize 150 --nomodel --call-summits --nolambda --keep-dup all -p 0.01”. (2) The summits of peaks were extended by 250 bp on both sides, then final width was 501 bp. (3) The regions of ENCODE hg38 blacklist (https://www.encodeproject.org/annotations/ENCFF356LFX/) were filtered out. (4) overlapping peaks within a single sample were removed using an iterative removal procedure keeping the most significant peaks based on MACS2 output’s ‘score’ values), identifying “a sample peak set”. (5) The values of “Score per million” were calculated by dividing each individual peak score by the sum of all peak scores in the each sample divided by 1 million. (6) The iterative removal procedure above was repeated across sample peak sets based on score per million. (7) The reproducible peak set was identified by selecting peaks with score per million ≥5 and overlaps between at least two samples, and peaks on chromosome Y were removed. Finally, we obtained a reproducible high-quality set of 501 bp fixed-width peaks for 42 ATAC-seq profiles. To get the number of Tn5 insertions in each peak, bam files were read as Genomic Ranges object in R using Rsamtool’s “scambam()” and corrected for Tn5 offset (“+” stranded +4 bp, “−” stranded −5 bp). Each corrected insertion was counted using “countOverlaps()”. The counts matrix was normalised by using edgeR’s “cpm(log = TRUE, prior.count = 5)” followed by a quantile normalisation using preprocessCore’s “normalize.quantiles()”. The width of each fragment was calculated, and fragments of less than 100 bp were selected as nucleosome-free fragments. We then identified a reproducible peak set and constructed a count matrix by the same procedure above using the nucleosome-free insertions.

### ATAC-seq data analysis—profiling peaks and tumours

To annotate peaks, ChIPseeker’s “annotatePeak()” function with default setting was used. The overlapping peaks between JFCR–BRCA peaks and TCGA–BRCA peaks were identified using “findOverlaps()”. To calculate correlations between tumours, “cor()” function with “method = ‘pearson’”. Promoter elements were the peaks annotated as “Promoter (≤1 kb)” by “annotatePeak()”, and any other peaks were defined as distal elements.

To get “distal cancer CREs”, promoter peaks and overlaps with TME-specific peaks (from ref. [28]) were removed by “findOverlaps(invert = TRUE)”. To classify ER + /HER2− tumours, hierarchical clustering (Ward’s minimum variance method) was performed by “hclust(distance, method = “ward.D2”)”. To classify distal cancer CREs, common accessible peaks were identified by the median accessibility and variance across tumours using “rowMedians()” and “rowVars()” (Median accessibility ≥ 3 and variance ≤ 0.5). After removing the common peaks, k-means clustering for top variable 50,000 peaks were performed by “kmeans(centers = 5, iter.max = 100)”.

### Differential analysis for ATAC-seq and RNA-seq data

R package edgeR’s glmQLFTest (v3.32.1) was used to identify differential accessible regions (DARs) for ATAC-seq and differential expressed genes (DEGs) for RNA-seq. Briefly, the library size normalisation, the dispersions estimation, and then the generalised linear model fitting were sequentially performed with’calcNormFactors(y, method = TMM),’estimateDisp(y, design = design, robust = TRUE)’, and’glmQLFit(y, design = design)’, respectively. Finally, log2 fold change and false discovery rates (FDR) of each region or gene between two groups were calculated by glmQLFTest. Regions with abs(log2FC) > 1 and FDR < 0.01 or genes with abs(log2FC) > 1 and FDR < 0.01 were identified as DARs or DEGs, respectively.

### Motif enrichment analysis—HOMER and ChromVAR

HOMER v4.10 “findMotifsGenome.pl” was used for motif enrichment analysis of each set of peaks with “-size 200 -mask -nomotif” options. Motif enrichment score was calculated by ChromVARs as follows: (i) adding GC bias information by “addGCBias()”, (ii) identifying elements with motifs by “matchMotifs()” using motif annotation of R package chromVARmotif’s “homer_pwms”, (iii) obtaining background peaks by “getBackgroundPeaks()”, (iv) calculating motif deviations by “computeDeviations()”. Z-scores of motif deviations (i.e. Motif scores) were used for analysis.

### Lisa Cistrome analysis for predicting transcriptional regulators

We calculated the numbers of nearby CREs of each gene based on the peak annotation by “annotatePeak()”. The genes with high numbers of nearby CREs were used for predicting upstream TFs as input for Lisa Cistrome (http://lisa.cistrome.org). Because the most gene number was restricted as 500 for Lisa input, we selected the genes below 500 genes.

### TCGA data analysis—ATAC-seq and RNA-seq

We downloaded TCGA chromatin accessibility profiles from National Cancer Institute Genomic Data Commons websites via browser (https://gdc.cancer.gov/about-data/publications/ATACseq-AWG). BRCA-specific normalised counts matrix and called peaks were used in this study (BRCA_log2norm.txt and BRCA_peakCalls.txt). We followed the clinical data presented by Supplementary Data 1 in ref. [25] for hormone receptor status and the Xena Functional Genomics Explorer TCGA Hub (https://xenabrowser.net/hub/) for histological subtypes. The analysis for TCGA data was performed using almost the same method of the analysis for JFCR–BRCA samples described above. TCGA–BRCA RNA-seq data as a SummarizedExperiment object was downloaded using R package ‘TCGAbiolinks”s “GDCquery(project = “TCGA–BRCA”, data.category = ”Transcriptome Profiling”, data.type = ”Gene Expression Quantification”, wokflow.type = ”STAR – Counts”)”, “GDCdownload()” and “GDCprepare()”. For survival analysis, we used “survival” package’s “survfit()” function and “survminer”s “ggsurvplot()” function.

### Overlap significant analysis

FOXA1 ChIP-seq data in breast cancer cell lines MCF-7 (ERX008600) and T47D (ERX008605) were downloaded from ChIP-Atlas (https://chip-atlas.org). ENCODE Transcription Factor Targets containing both of ESR1 and FOXA1 target genes were downloaded from Harmonizome website (https://maayanlab.cloud/Harmonizome/dataset/ENCODE+Transcription+Factor+Targets). To calculate overlap significant between each peak set, we utilised Bedtools’s fisher function.

## Supplementary information

Supplementary Figures

Supplementary Tables

## Data Availability

Processed ATAC-seq data have been deposited at GEO (GSE222116) and are publicly available.

## Code availability

R code for reproducing the analyses is available at https://github.com/KoheiKumegawa/CA_JFCR_BRCA.
