# Correction: Chromatin profile-based identification of a novel ER-positive breast cancer subgroup with reduced ER-responsive element accessibility

**DOI:** 10.1038/s41416-023-02206-0

**Published:** 2023-03-13

**Authors:** Kohei Kumegawa, Sumito Saeki, Yoko Takahashi, Liying Yang, Tomo Osako, Tomoyoshi Nakadai, Sayuri Amino, Tetsuyo Maeda, Chikako Takahata, Seiichi Mori, Tetsuo Noda, Shinji Ohno, Takayuki Ueno, Reo Maruyama

**Affiliations:** 1 Cancer Cell Diversity Project, NEXT-Ganken Program, Japanese Foundation for Cancer Research, Tokyo, Japan; 2 Breast Surgical Oncology, Breast Oncology Center, Cancer Institute Hospital, Japanese Foundation for Cancer Research, Tokyo, Japan; 3 Project for Cancer Epigenomics, Cancer Institute, Japanese Foundation for Cancer Research, Tokyo, Japan; 4 Division of Pathology, Cancer Institute, Japanese Foundation for Cancer Research, Tokyo, Japan; 5 Project for Development of Innovative Research on Cancer Therapeutics, Cancer Precision Medicine Center, Japanese Foundation for Cancer Research, Tokyo, Japan; 6 Director’s room, Cancer Institute, Japanese Foundation for Cancer Research, Tokyo, Japan; 7 Breast Oncology Center, Cancer Institute Hospital, Japanese Foundation for Cancer Research, Tokyo, Japan

**Keywords:** Breast cancer, Breast cancer

Correction to: *British Journal of Cancer* 10.1038/s41416-023-02178-1, published online 01 February 2023

The original version of this article unfortunately contained errors in figures. After publication, the authors found that the downloaded version of PDF has several distorted figures. This is due to an error in typesetting. The list of distorted figures is:


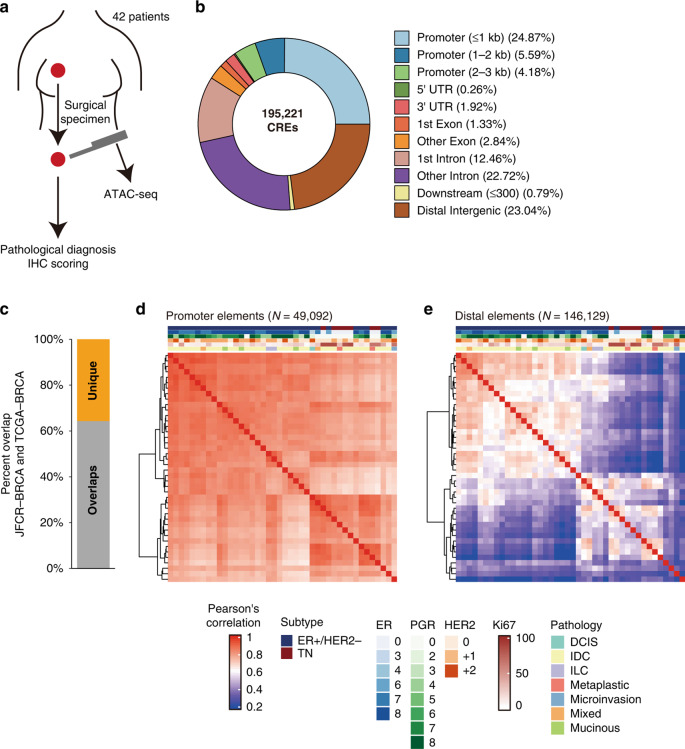
Figure 1d-e: The heatmaps are distorted and lack some annotation panels (top of heatmaps)


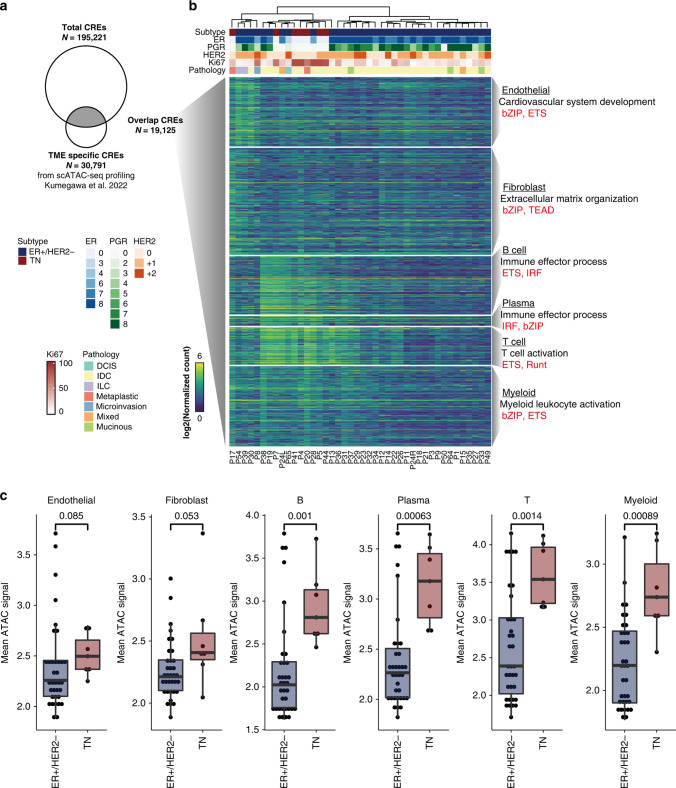
Figure 2b: The heatmap lacks some annotation panels (top of heatmap, “Subtype” annotation)


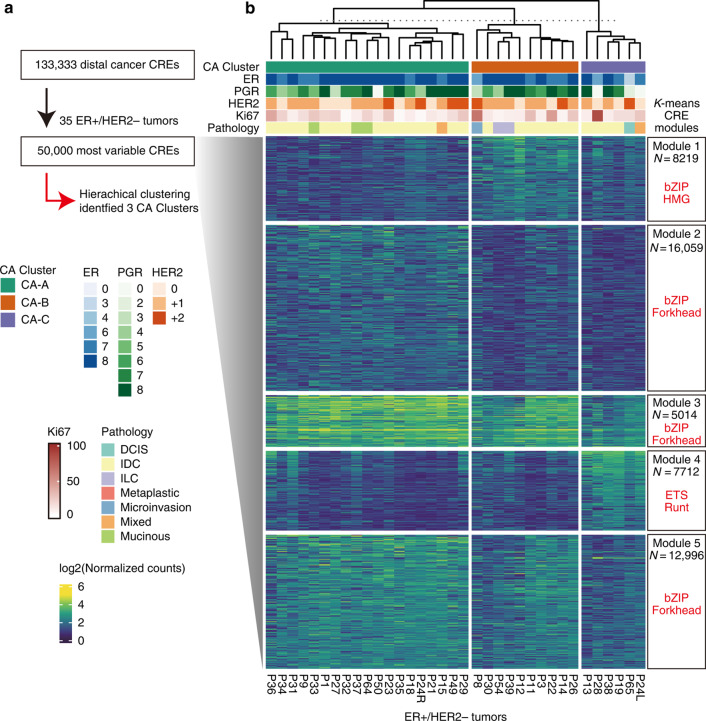
Figure 4b: The heatmap lacks some annotation panels (top of heatmap, “CA Cluster” annotation)


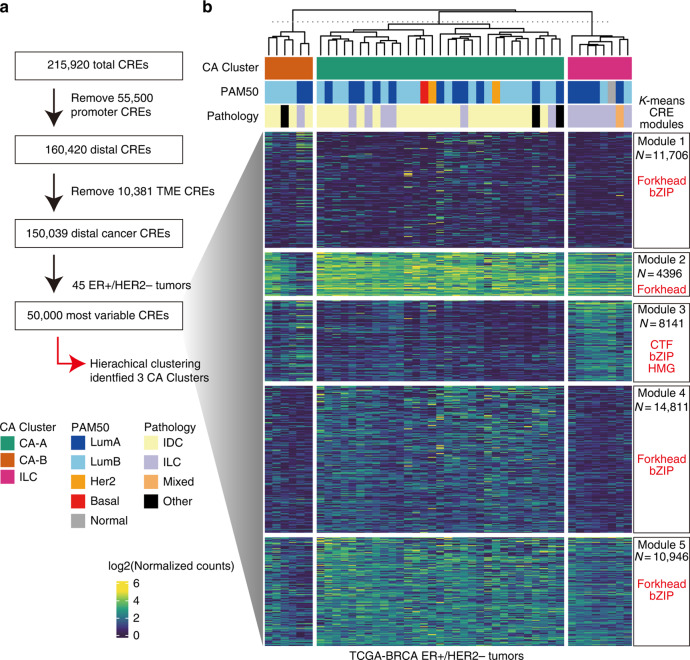
Figure 7b: The heatmap lacks some annotation panels (top of heatmap, “CA Cluster” annotation)

The correct figures can be found above. The original article has been corrected.

