# Supplementary material for: Chromatin profile-based identification of a novel ER-positive breast cancer subgroup with reduced ER-responsive element accessibility
Source: Br J Cancer. 2023 Feb 1;128(7):1208–22. doi: 10.1038/s41416-023-02178-1 (PMC10050410; doi:10.1038/s41416-023-02178-1)
Supplement: Supplementary file 1 — Supplementary Figures [file 41416_2023_2178_MOESM1_ESM.pdf]

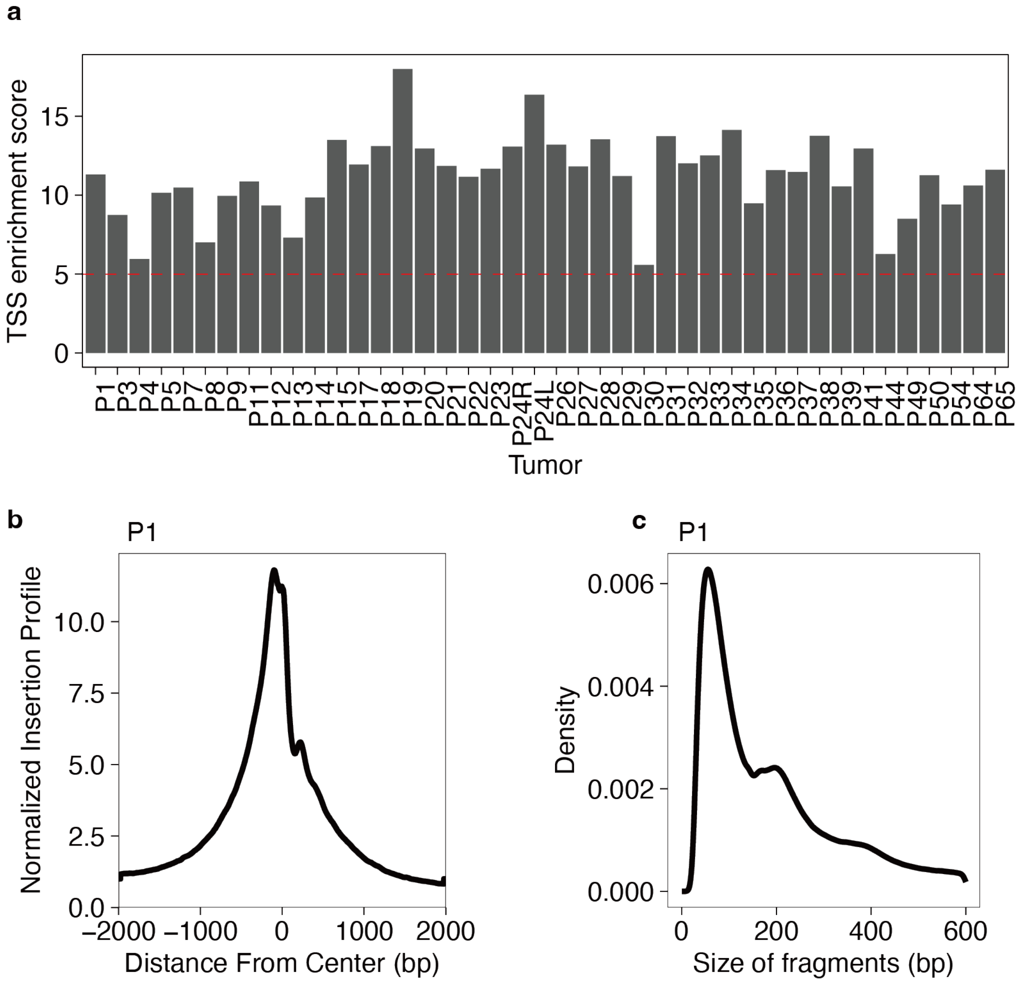

Supplementary Figure 1. ATAC-seq quality

(a) Bar plot representing TSS enrichment score of each sample. (b–c) Example of normalized insertion profile in (b) and fragment size distribution in (c) (P1 data is presented).

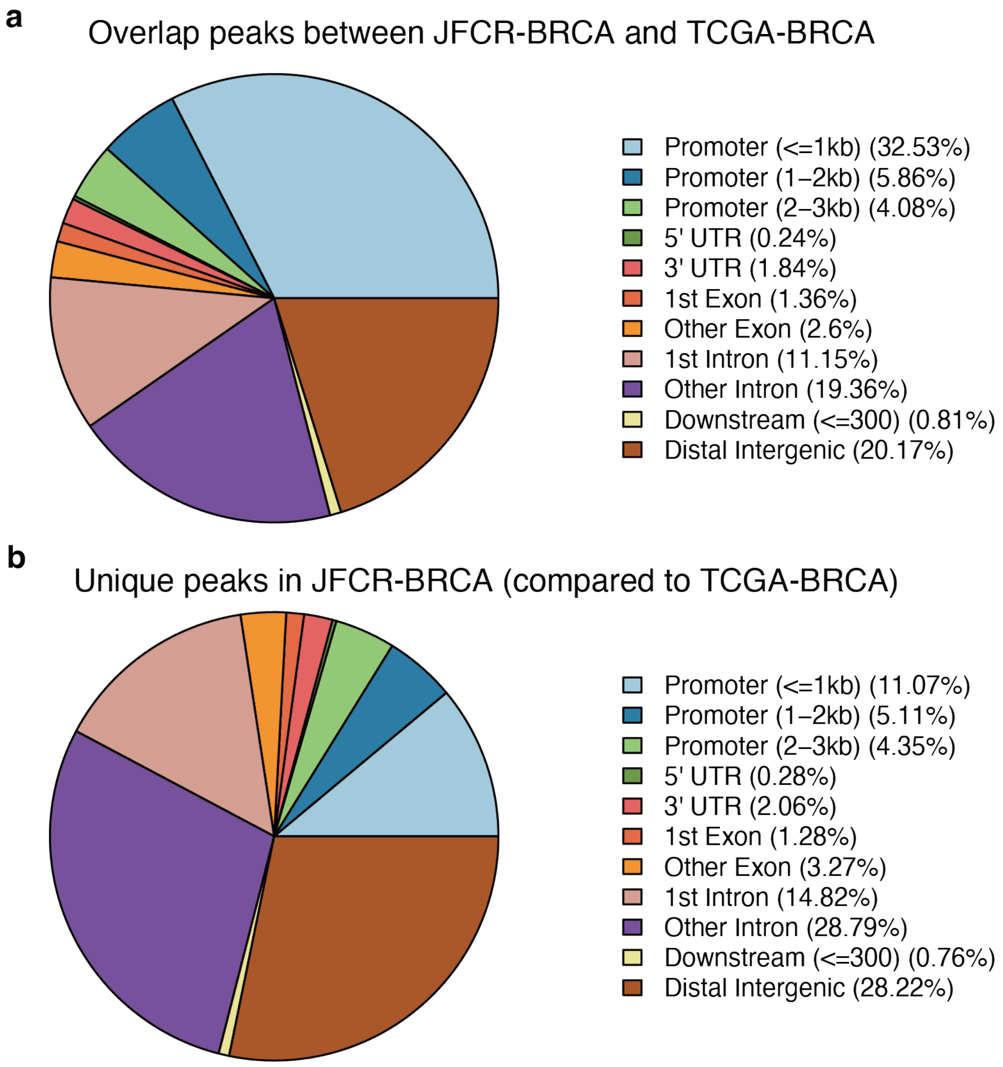

Supplementary Figure 2. Genomic features of the overlapping peaks and unique peaks between JFCR-BRCA and TCGA-BRCA ATAC-seq

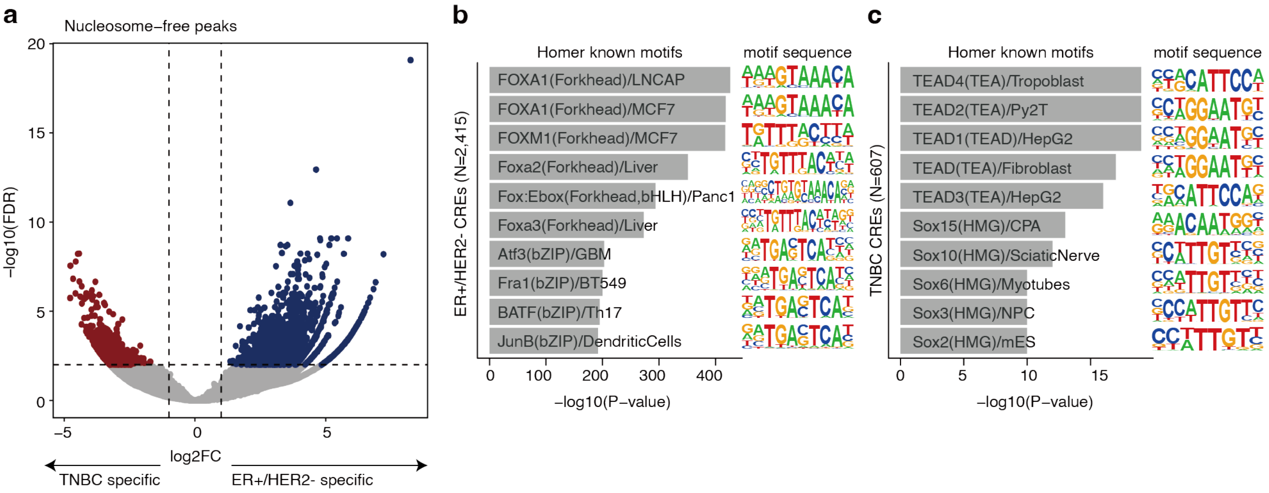

Supplementary Figure 3. Differential accessible analysis of nucleosome-free peaks between ER+/HER2- tumors and TNBC.

(a) Volcano plot showing differential accessibility analysis for nucleosome-free peaks between ER+/HER2- and TNBC. Significantly different CREs were colored by red (TNBC-specific) or blue (ER+/HER2- specific). (b, c) Bar plot of motif enrichment significance (P-value) of Homer known motifs for ER+/HER2- specific peaks (b) and TNBC-specific peaks (c). Shown on right are known motif sequences.

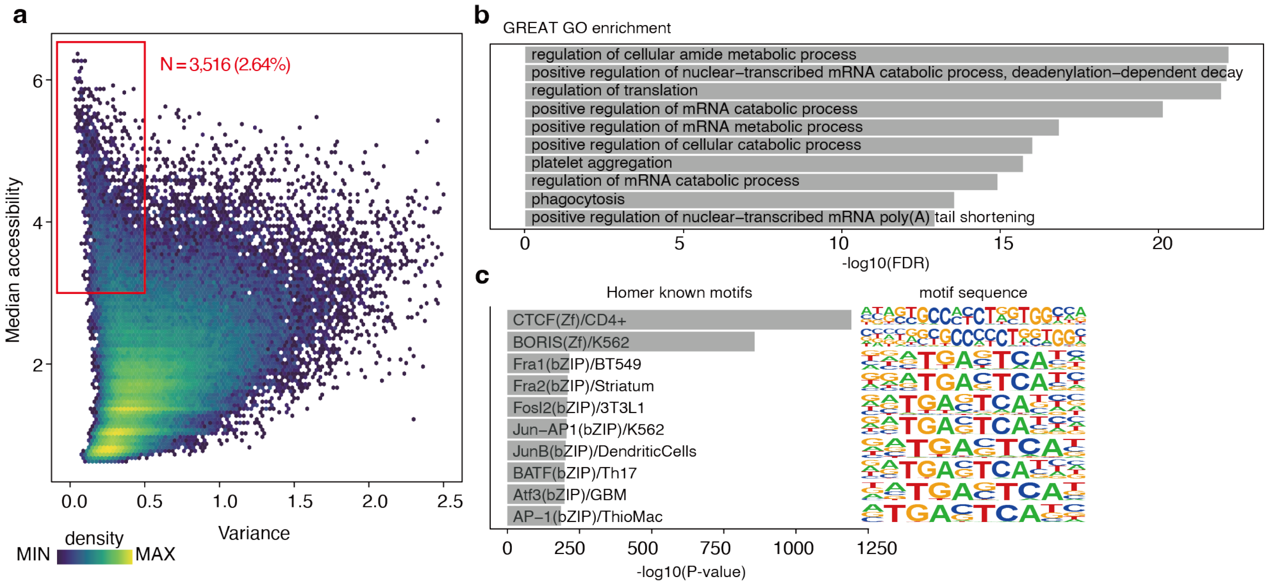

Supplementary Figure 4. Commonly accessible CREs in ER+/HER2- tumors

(a) Density map comparing the median and variance of chromatin accessibility at each CRE across tumors. Each dot represents a CRE. Red box indicates CREs with relatively high ATAC-seq signals and low variability across tumors (termed “common accessible CREs”). (b) Bar plot of gene ontology enrichment using GREAT analysis for commonly accessible CREs. (c) Bar plot of motif enrichment significance (P-value) of Homer known motifs for commonly accessible CREs. Shown on right are known motif sequences.

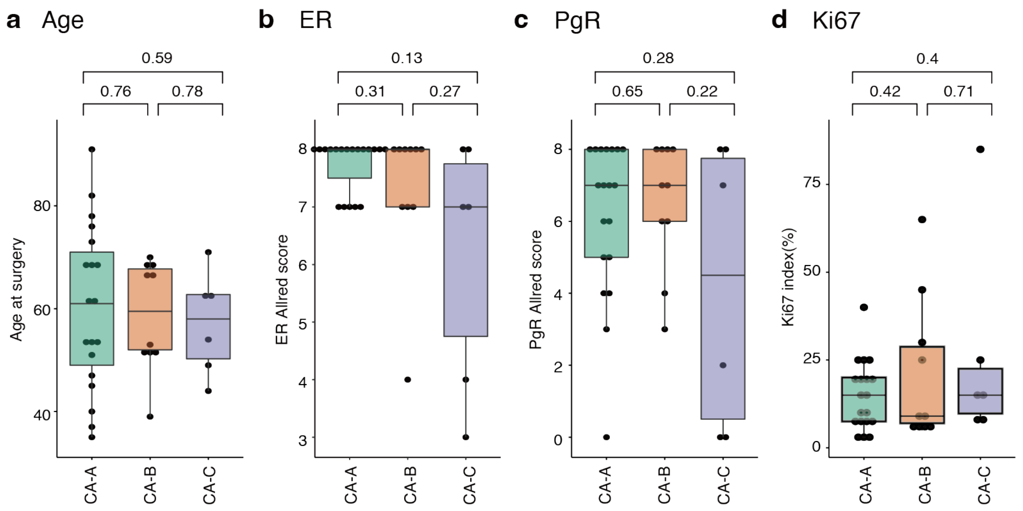

Supplementary Figure 5. Clinical features and chromatin accessibility clusters

Box plots representing age (a), ER Allred IHC score (b), PgR Allred IHC score (c), and Ki67 index (d). P-values calculated by Student' s t test are shown.

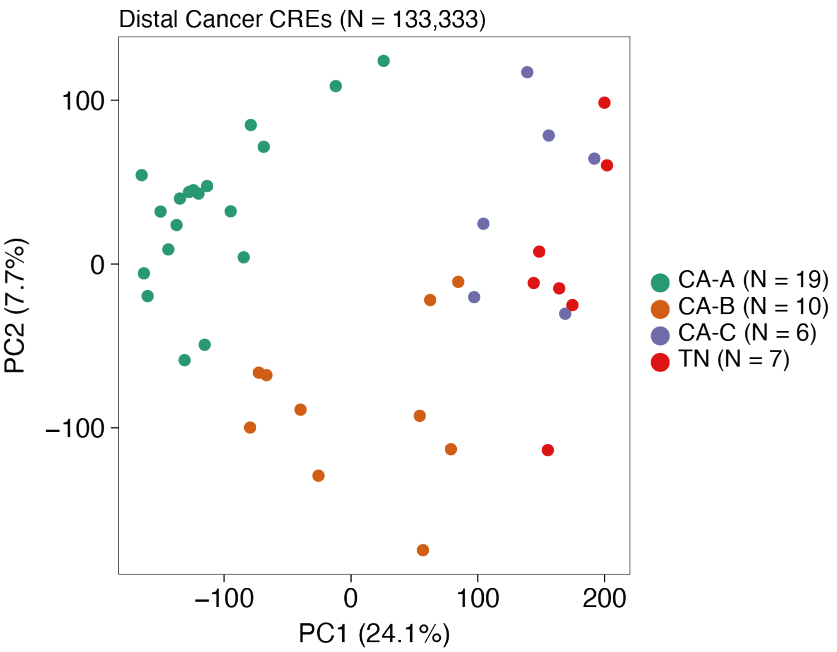

Supplementary Figure 6. Principal component analysis of 42 JFCR-BRCA tumors.

Dot plots showing PCA using distal cancer CREs (N=133,333). Each dot represents each tumor colored by chromatin accessibility clusters (ER+/HER2-) or TNBC.

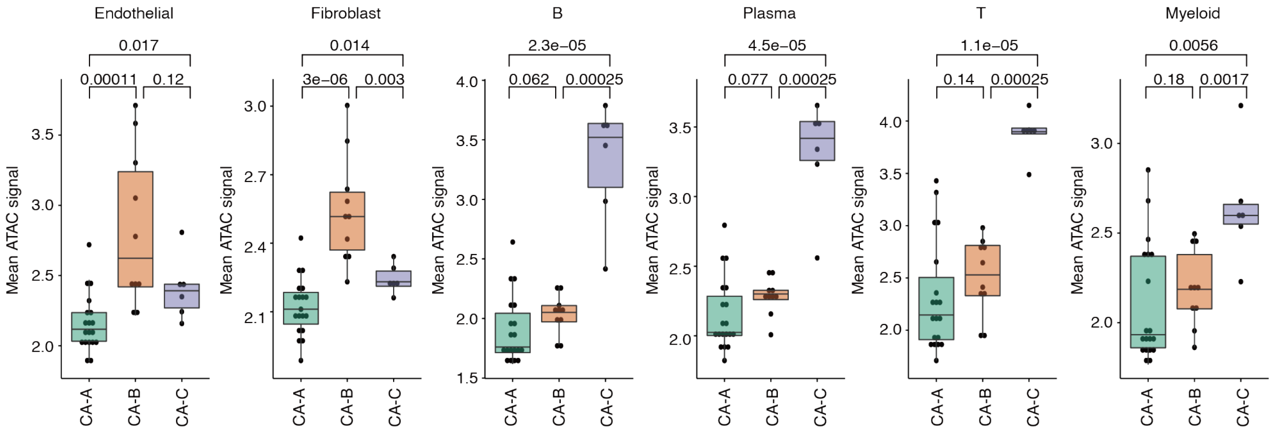

Supplementary Figure 7. Accessibility difference of TME specific CREs between chromatin clusters. Box plot showing mean ATAC-seq signal of each set of TME specific CREs between chromatin accessibility clusters. P-values calculated by Wilcoxon rank sum test are shown.

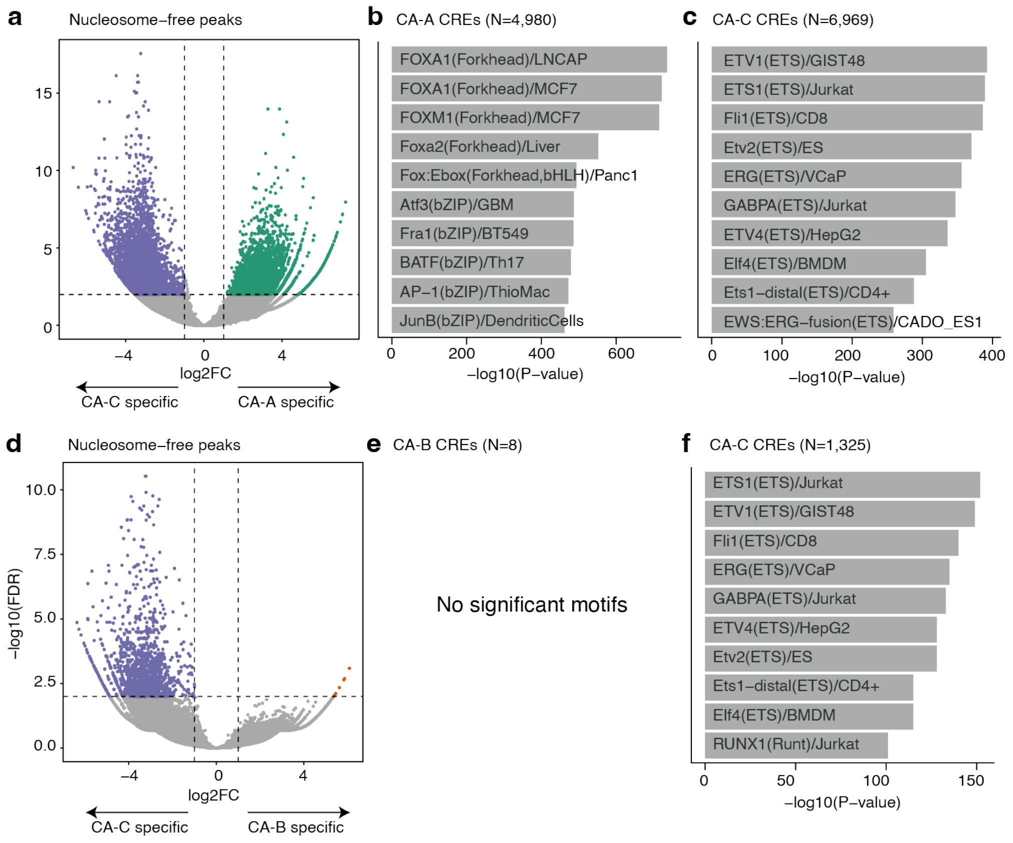

Supplementary Figure 8. Differential accessible analysis of nucleosome-free peaks between CA-C and CA-A or CA-C and CA-B.

(a) Volcano plot showing differential accessibility analysis for nucleosome-free peaks between CA-C and CA-A tumors. Significantly different CREs were colored by according to each cluster corresponding to Figure 4b. (b, c) Bar plot of motif enrichment significance (P-value) of Homer known motifs for CA-A-specific peaks (b) and CA-C-specific peaks (c). (d) Volcano plot showing differential accessibility analysis for nucleosome-free peaks between CA-C and CA-B tumors. Significantly different CREs were colored by according to each cluster corresponding to Figure 4b. (e, f) Bar plot of motif enrichment significance (P-value) of Homer known motifs for CA-B-specific peaks (no enriched motifs were detected) and CA-C-specific peaks (f).

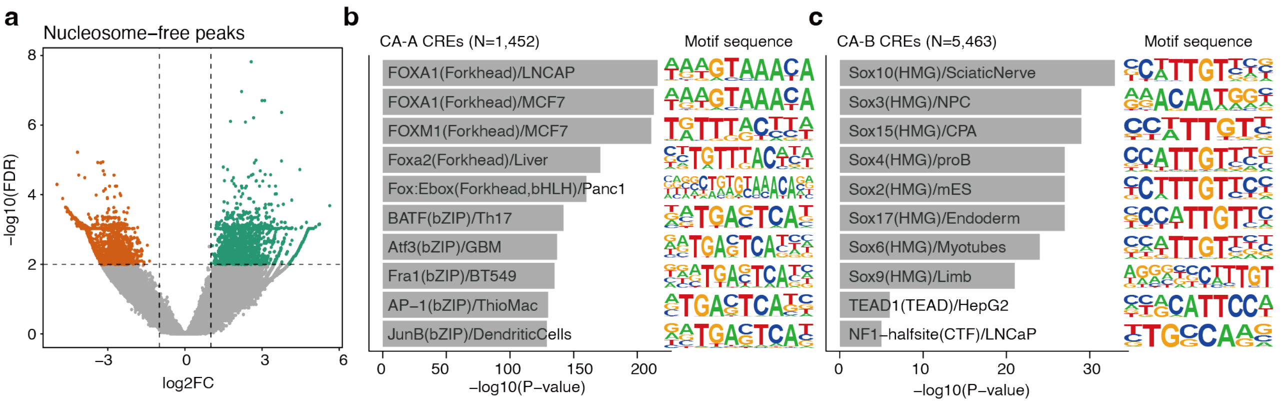

Supplementary Figure 9. Differential accessible analysis of nucleosome-free peaks between CA-A and CA-B.

(a) Volcano plot showing differential accessibility analysis for nucleosome-free peaks between CA-A and CA-B tumors. Significantly different CREs were colored by according to each cluster corresponding to Figure 4b. (b, c) Bar plot of motif enrichment significance (P-value) of Homer known motifs for CA-A-specific peaks (b) and CA-C-specific peaks (c). Shown on right are known motif sequences.

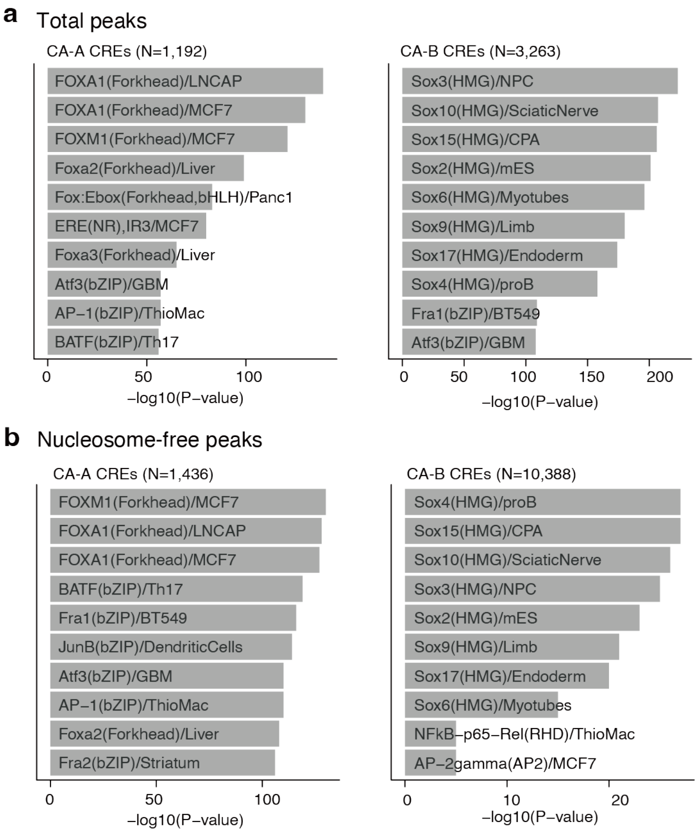

Supplementary Figure 10. Differential accessible analysis between IDC CA-A and IDC CA-B tumors.
(a) Bar plot of motif enrichment significance (P-value) of Homer known motifs for CA-A-specific peaks (left) and CA-B-specific peaks (right). (b) Bar plot of motif enrichment significance (P-value) of Homer known motifs for CA-A-specific nucleosome-free peaks (left) and CA-B-specific nucleosome-free peaks (right).

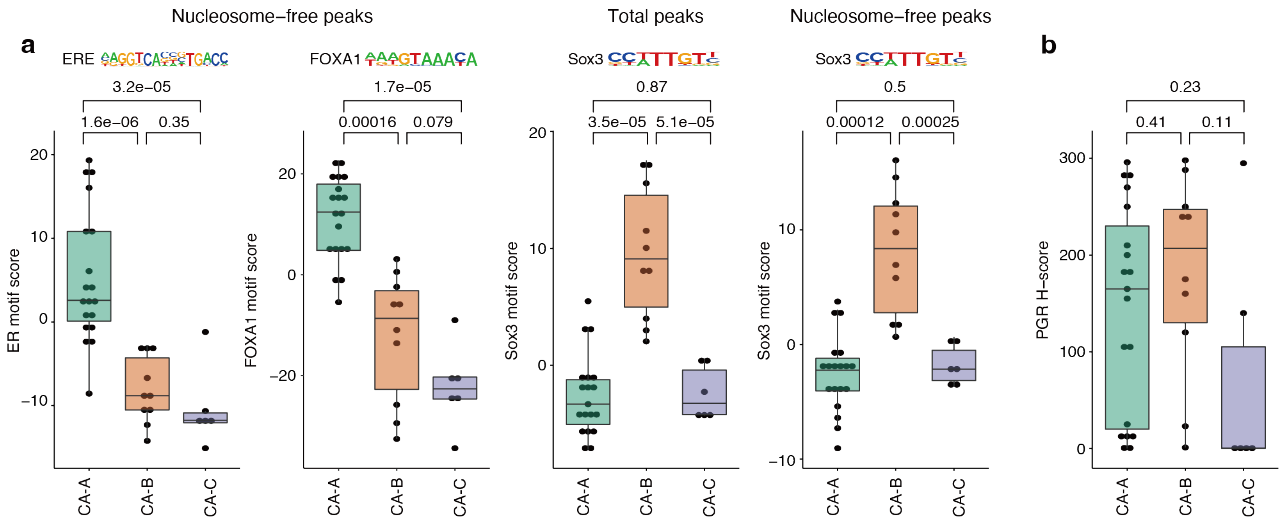

Supplementary Figure 11. Motif enrichment score of key TFs and PGR H-score

(a) Box plot representing ER and FOXA1 motif score calculated using nucleosome-free peaks, and Sox3 motif score calculated by total peaks and nucleosome-free peaks. Motif sequences are shown on top of the box plots. P-values calculated by Student' s t test are shown. (b) Box plot representing PGR H-score of each chromatin cluster.

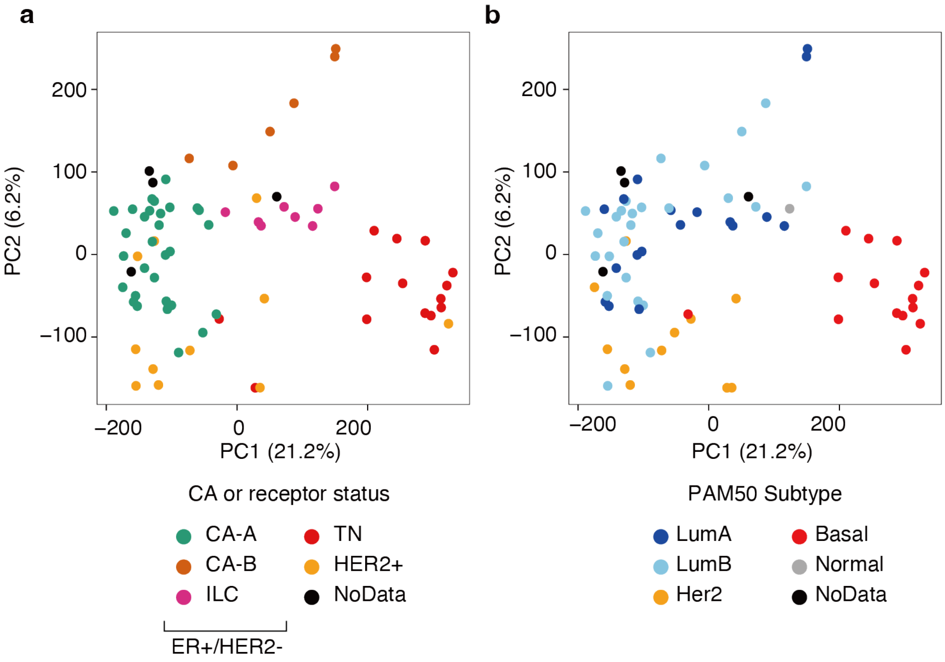

Supplementary Figure 12. Principal component analysis of 75 TCGA-BRCA tumors.

Dot plots showing PCA. Each dot represents each tumor colored by chromatin accessibility clusters (ER+/HER2-) or receptor status (a); and colored by PAM50 classification based on RNA expression (b).

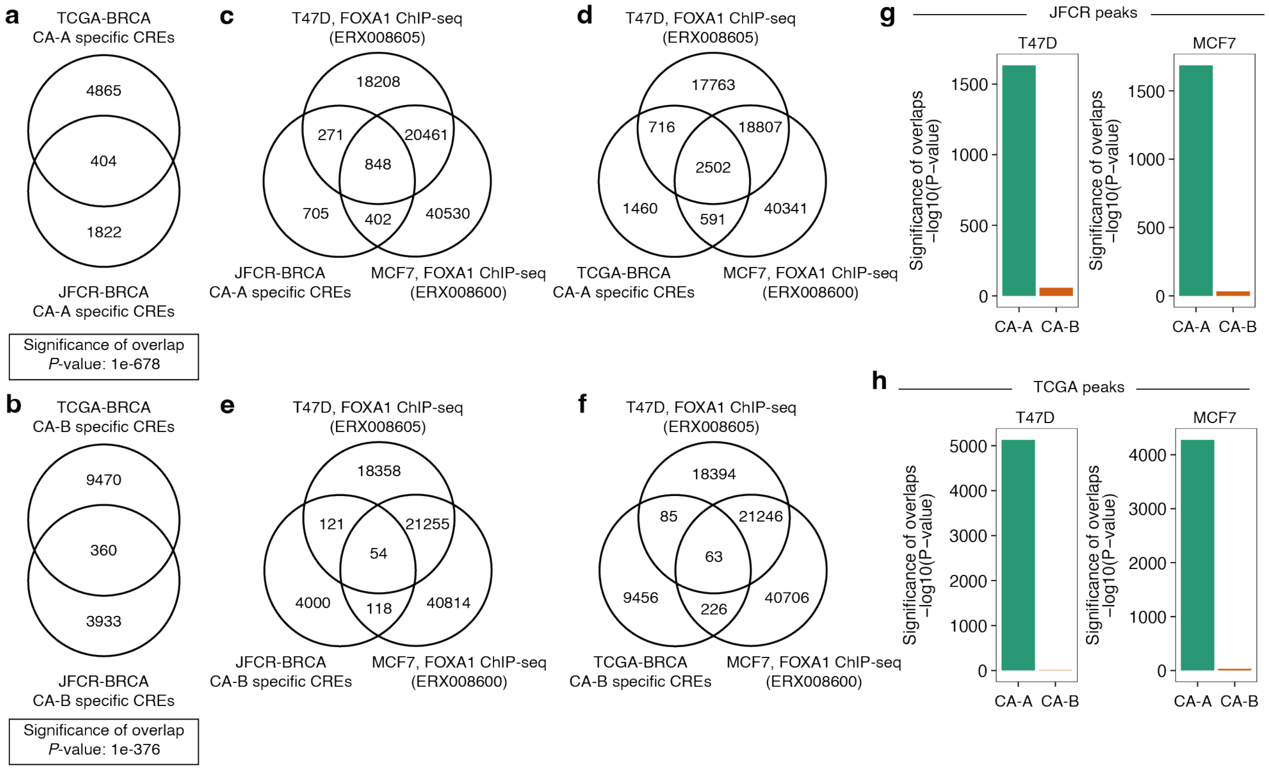

Supplementary Figure 13. Overlaps between CA-A/CA-B-specific CREs and publicly available FOXA1 binding sites in luminal cell lines.

Venn plot showing overlaps (a) between JFCR-BRCA CA-A-specific CREs and TCGA-BRCA CA-A-specific peaks, (b) between JFCR-BRCA CA-B-specific CREs and TCGA-BRCA CA-B-specific peaks, (c) between JFCR-BRCA CA-A specific CREs and FOXA1 ChIP-seq peaks in luminal breast cell lines T47D and MCF7, (d) between TCGA-BRCA CA-A specific CREs and FOXA1 ChIP-seq peaks in luminal breast cell lines T47D and MCF7, (e) between JFCR-BRCA CA-B specific CREs and FOXA1 ChIP-seq peaks in luminal breast cell lines T47D and MCF7, and (f) between TCGA-BRCA CA-B specific CREs and FOXA1 ChIP-seq peaks in luminal breast cell lines T47D and MCF7. (g–h) Significance of overlaps between JFCR CA-A or CA-B peaks and FOXA1 ChIP-seq in (g), and between TCGA CA-A or CA-B peaks and FOXA1 ChIP-seq in (h). All overlap significances are calculated by fisher’ s exact test using ‘Bedtools fisher’ function.

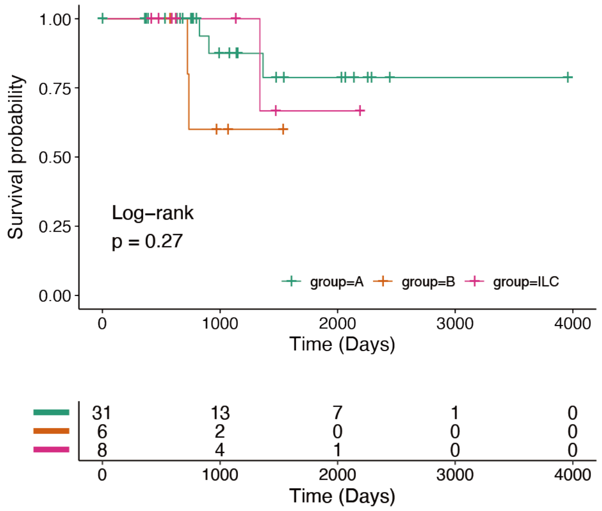

Supplementary Figure 14. Survival analysis for TCGA-BRCA 45 ER+/HER2- tumors stratified by chromatin accessibility clusters.
Kaplan-Meier survival curve. Censored values (+) represent the last known follow-up time. P-value is calculated by a log-rank test.
